# PbrChiA: a key chitinase of pear in response to *Botryosphaeria dothidea* infection by interacting with PbrLYK1b2 and down-regulating ROS accumulation

**DOI:** 10.1093/hr/uhad188

**Published:** 2023-09-19

**Authors:** Qiming Chen, Huizhen Dong, Qionghou Li, Xun Sun, Xin Qiao, Hao Yin, Zhihua Xie, Kaijie Qi, Xiaosan Huang, Shaoling Zhang

**Affiliations:** State Key Laboratory of Crop Genetics and Germplasm Enhancement, Centre of Pear Engineering Technology Research, Nanjing Agricultural University, Nanjing 210095, China; State Key Laboratory of Crop Genetics and Germplasm Enhancement, Centre of Pear Engineering Technology Research, Nanjing Agricultural University, Nanjing 210095, China; State Key Laboratory of Crop Genetics and Germplasm Enhancement, Centre of Pear Engineering Technology Research, Nanjing Agricultural University, Nanjing 210095, China; State Key Laboratory of Crop Genetics and Germplasm Enhancement, Centre of Pear Engineering Technology Research, Nanjing Agricultural University, Nanjing 210095, China; State Key Laboratory of Crop Genetics and Germplasm Enhancement, Centre of Pear Engineering Technology Research, Nanjing Agricultural University, Nanjing 210095, China; State Key Laboratory of Crop Genetics and Germplasm Enhancement, Centre of Pear Engineering Technology Research, Nanjing Agricultural University, Nanjing 210095, China; State Key Laboratory of Crop Genetics and Germplasm Enhancement, Centre of Pear Engineering Technology Research, Nanjing Agricultural University, Nanjing 210095, China; State Key Laboratory of Crop Genetics and Germplasm Enhancement, Centre of Pear Engineering Technology Research, Nanjing Agricultural University, Nanjing 210095, China; State Key Laboratory of Crop Genetics and Germplasm Enhancement, Centre of Pear Engineering Technology Research, Nanjing Agricultural University, Nanjing 210095, China; State Key Laboratory of Crop Genetics and Germplasm Enhancement, Centre of Pear Engineering Technology Research, Nanjing Agricultural University, Nanjing 210095, China

## Abstract

Pear ring rot, caused by the pathogenic fungi *Botryosphaeria dothidea*, seriously affects pear production. While the infection-induced reactive oxygen species (ROS) burst of infected plants limits the proliferation of *B. dothidea* during the early infection stage, high ROS levels can also contribute to their growth during the later necrotrophic infection stage. Therefore, it is important to understand how plants balance ROS levels and resistance to pathogenic *B. dothidea* during the later stage. In this study, we identified *PbrChiA*, a glycosyl hydrolases 18 (GH18) chitinase-encoding gene with high infection-induced expression, through a comparative transcriptome analysis. Artificial substitution, stable overexpression, and virus induced gene silencing (VIGS) experiments demonstrated that PbrChiA can positively regulate pear resistance as a secreted chitinase to break down *B. dothidea* mycelium *in vitro* and that overexpression of *PbrChiA* suppressed infection-induced ROS accumulation. Further analysis revealed that PbrChiA can bind to the ectodomain of PbrLYK1b2, and this interaction suppressed PbrLYK1b2-mediated chitin-induced ROS accumulation. Collectively, we propose that the combination of higher antifungal activity from abundant PbrChiA and lower ROS levels during later necrotrophic infection stage confer resistance of pear against *B. dothidea*.

## Introduction

Plants have an innate immune system that can detect and respond to external threats by recognizing pathogen-associated molecular patterns (PAMPs) [1]. Many cell surface receptors are responsible for identifying extracellular non-self-substances, and PAMPs typically exhibit significant levels of conservation and fulfill crucial roles across the entire microbial class, such as chitin, β-glucans, and flagellin [2]. When plants recognize PAMPs, it triggers a response known as PAMP-triggered immunity (PTI), which includes the production of reactive oxygen species (ROS), ion flux, transcriptional reprogramming, and the synthesis of new proteins that work together to restrict the growth of pathogens [3, 4].

Chitin is a potent PAMP found in the fungal cell wall, and chitin oligomers can induce PTI in various plant species, causing local ROS bursts and activating related enzymes such as SOD, POD, and chitinase, among others [5]. Chitin elicitor receptor kinase1 (CERK1) is a well-studied plant pattern recognition receptor (PRR) with extracellular chitin-binding lysin-motifs (LysM) that activate chitin signaling [6–9]. Within PTI responses, ROS often play a pivotal role in successful disease resistance responses in plants. The host’s local hypersensitive responses (HR) with high-level ROS led to cell death and limit the proliferation of pathogens in the early infection stage. However, certain pathogens have evolved strategies to manipulate ROS production for their own benefit. For instance, some fungal pathogens have evolved to mitigate chitin-triggered immunity due to the high selection pressure caused by the arms race with the plant immune system [10], including using LysM domain-containing effectors or enzymatically inactive chitinase to sequester chitin fragments from damaged cell walls, thereby suppressing chitin-induced PTI in host plants [2, 11, 12].

Pear ring rot is one of the main diseases affecting pear production in China, caused by the necrotrophic pathogenic fungus *Botryosphaeria dothidea*. This fungus causes local proliferation and wart formation on branches and stems, leading to the ring necrosis cell death spot around the infection site on infected shoots, stems, and fruits [13]. *B. dothidea* are known to lead to cell death which contributes to their growth in the later necrotrophic stage [14, 15]. ROS production is a multifaceted signaling mechanism in plants, mediating a range of responses that can either be beneficial or detrimental. Therefore, balancing ROS levels and resistance during the necrotrophic infection stage is essential for optimal plant defense against pathogenic *B. dothidea*.

In this study, we conducted a comparative transcriptome analysis and identified PbrChiA, a member of the GH18 family chitinase, as a key regulator in enhancing pear resistance to *B. dothidea*. Our results showed that PbrChiA functions as a secreted chitinase that directly exerts anti-fungal activity while also inhibiting chitin-induced ROS accumulation by interacting with PbrLYK1b2, a homolog of AtCERK1. We propose that higher antifungal activity resulting from abundant PbrChiA and lower ROS levels during later necrotrophic stages combine to confer resistance of pear against *B. dothidea*.

## Results

### Phenotype identification of susceptible S-589 and resistant R-532 in response to *B. dothidea* infection

**Figure 1:**
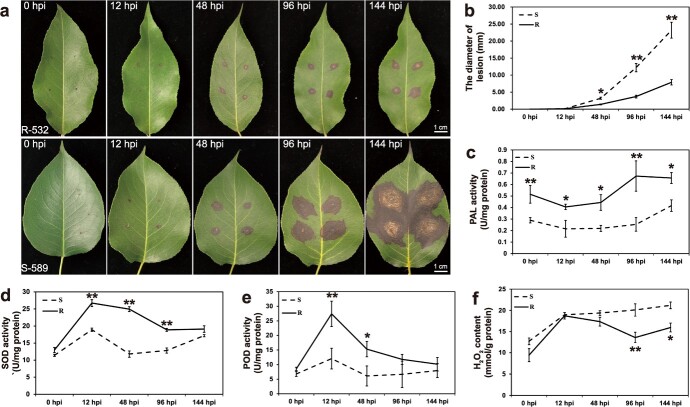
The phenotypes and physiological indicators of resistant cultivar R-532 and susceptible cultivar S-589 infected by *Botryosphaeria dothidea*. (**a**) Phenotypes of resistant cultivar R-532 and susceptible cultivar S-589 infected by *B. dothidea*. With the infection of *B. dothidea*, resistant cultivar R-532 showed greater disease resistance. (**b**) Measurement of the lesion diameters of the R-532 and S-589. The diameters of infection lesion on S-589 were larger than on R-532. (**c**–**e**) Measurement of PAL (**c**), SOD (**d**) and POD (**e**) activities of R-532 and S-589. (**f**) Measurement of H_2_O_2_ content of R-532 and S-589. Data are shown as mean ± SD. At least three biological repetitions were performed. The data were analysed using two-way ANOVA followed by Sidak’s test. ^**^P < 0.01, ^*^P < 0.05.

To understand the molecular basis of highly variable disease resistance among pear cultivars, we selected two Chinese white pear cultivars, S-589 and R-532, as susceptible and resistant genotypes, respectively, to perform *B. dothidea* infection treatment (Fig. 1). We found that the lesions of S-589 grew more severely and expanded faster than those of R-532, as depicted in Fig. 1a and b. However, there was no significant difference in lesion diameter between the two varieties until 48 hpi. To study the physiological and biochemical responses to *B. dothidea* infection, we monitored them from 0 to 144 hpi. Under non-treated conditions, S-589 and R-532 exhibited similar levels of superoxide dismutase (SOD) and peroxidase (POD) activities, and hydrogen peroxide (H_2_O_2_) contents, except for phenylalanine ammonia lyase (PAL) activity, which was approximately 1.7 times higher in R-532 than in S-589 (Fig. 1c–f).

Although there was no significant difference in lesion diameters, the activities of SOD and POD, and H_2_O_2_ contents were elevated in both S-589 and R-532 at 12 hpi (hours post infection), indicating strong activation of immune responses during the early infection stage before the lesion formation (Fig. 1d–f). Despite having stronger defense-related reactions with higher related enzyme activities than S-589, different levels of lesion formation and development were observed in the leaves of the two genotypes at 48hpi, suggesting that the different phenotypes between R-532 and S-589 were mainly from the later infection stages.

From 48 to 144 hpi, with the formation and expansion of the lesions, the activities of SOD and POD in R-532 began to decrease, while those in S-589 showed a slow rising trend. Notably, during the entire infection treatment, R-532 showed higher activities of SOD, POD, PAL, and lower H_2_O_2_ contents than those measured in S-589 (Fig. 1c–f). The different phenotypes in *B. dothidea* resistance between R-532 and S-589 may be related to the activation and regulation of resistance associated genes. Based on changes in physiological indicators after *B. dothidea* infection, we collected samples from the early and later infection stages for the following sequencing analysis.

### RNA-seq data processing and read mapping

RNA-seq technology was employed to identify important genes related to resistance in susceptible S-589 and resistant R-532 leaves under *B. dothidea* infection. A total of 30 cDNA libraries were constructed, consisting of two experimental groups: S_mock, S_12, S_48, S_96, S_144 and R_mock, R_12, R_48, R_96, R_144. The quality of the cDNA libraries is presented in Table S1 (see online supplementary material). The raw read count and mapping rate across these libraries ranged from 69 to 94 million and 69.29% to 77.87%, respectively, with a uniquely mapping rate of approximately 67%. Furthermore, the mean Q30 score, which indicates sequencing base calls with an error rate of less than 0.1%, was greater than 93%. These findings suggest that the overall quality of the transcriptome sequencing data was sufficiently high to support subsequent analyses (Table S1, see online supplementary material).

### Comparative transcriptomic analysis of susceptible S-589 and resistant R-532 in response to *B. dothidea* infection

**Figure 2:**
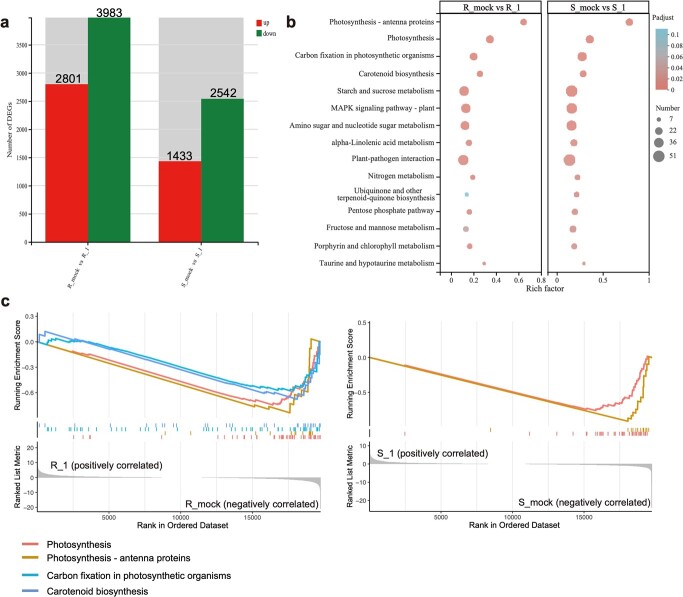
Gene expression analyses of early DEGs. **(a**) Numbers of early DEGs at 12 hpi in resistant cultivar R-532 and susceptible cultivar S-589. The first set of numbers represents the number of genes upregulated after *Botryosphaeria dothidea* infection, and the second set indicates the number of genes downregulated after *B. dothidea* infection. (**b**) KEGG functional enrichment analysis of early DEGs in R-532 and S-589. (**c**) Gene Set Enrichment Analysis of early DEGs in R-532 and S-589.

According to above results, the infection process can be divided into two main stages. The early stage occurs between 0 hpi to 12 hpi, before lesion formation. The later stage occurs between 48 and 144 hpi, during which successful infection and expansion of *B. dothidea* takes place. Compared to untreated controls, S-589 and R-532 exhibited 3975 (1433 genes up-regulated and 2542 genes down-regulated) and 6784 (2801 genes up-regulated and 3983 genes down-regulated) differentially expressed genes (DEGs), respectively, in the early stage (Fig. 2a; Table S2, see online supplementary material).

KEGG enrichment analysis (Fig. 2b; Table S3, see online supplementary material) revealed that DEGs from both genotypes were mainly enriched in similar pathways associated with photosynthesis (ko00195 and ko00196), carbon fixation (ko00710), ABA biosynthesis (ko00906), as well as plant-pathogen interaction (ko04626) and MAPK signaling (ko04016) pathways. To understand the changing pattern of these DEGs, a gene set enrichment analysis (GSEA) was performed. The GSEA showed that most of the genes from photosynthesis-associated pathways (ko00195 and ko00196) were down-regulated in both genotypes upon infection treatment, indicating that plant photosynthesis was inhibited after being infected by pathogens (Fig. 2c). In contrast to S-589, a significant down-regulation of genes in carbon fixation in photosynthetic organisms and ABA biosynthesis-related pathways was observed in R-532, suggesting that R-532 may allocate more energy towards adapting to the pathogen infection.

**Figure 3:**
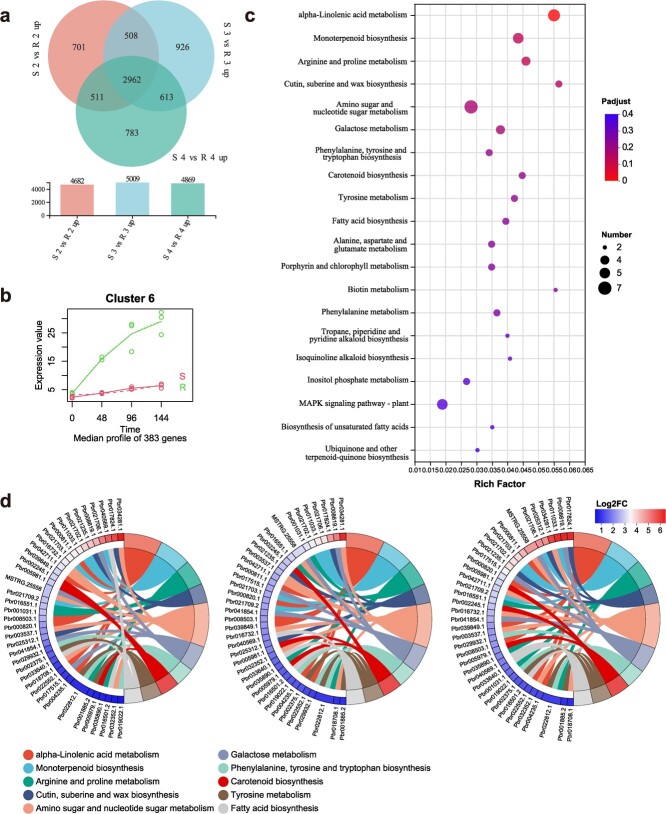
Gene expression analyses of later DEGs. (**a**) Venn diagram showing the numbers and overlap between high-expressed DEGs from three ‘Susceptible vs Resistant’ comparisons. (**b**) Expression pattern diagram of Cluster 6 from time-course differential expression analysis. The uper line indicates the genes expression pattern of R-532, and the lower line indicates the genes expression pattern of S-589. (**c**) KEGG functional enrichment analysis of later DEGs in R-532 and S-589. (**d**) The chordal graphs of Cluster 6 genes at three later infection stages. The three chordal graphs from left to right represent 48 hpi, 96 hpi, and 144 hpi, respectively. The left hemisphere indicates the changing fold of enriched genes, and the right hemisphere indicates the top ten enriched KEGG pathways.

To investigate the mechanisms underlying the contrasting resistance between R-532 and S-589 at later stages, a comparative analysis was conducted between ‘Susceptible vs Resistant’. From this, 2962 genes were identified as relatively high-expressed DEGs, which were then utilized to perform a time-course differential expression analysis via R::maSigPro (Fig. 3a; Table S2, see online supplementary material). As shown in Fig. 3b, 383 DEGs of Cluster 6 were selected, which were specifically highly continuously expressed in R-532 after *B. dothidea* infection (Fig. S1 and S2, see online supplementary material). This differential expression indicated that these genes might be involved in conferring resistance of R-532 to *B. dothidea* infection. KEGG enrichment and the chordal graph of Cluster 6 helped identify a GH18 family gene, *PbrChiA* (*Pbr018708.1*), from the ‘Amino sugar and nucleotide sugar metabolism’ pathway encoding a chitinase (EC3.2.1.14), that displayed high expression levels in R-532 across all three comparisons (Fig. 3c–d; Table S4, see online supplementary material).

Furthermore, HMM-based screening retrieved a total of 31 GH18 family genes from the Chinese white pear genome (Fig. S3a; Table S5, see online supplementary material). The resulting expression heatmap indicates that *PbrChiA* was strongly and consistently induced during later *B. dothidea* infection stages in R-532 (Fig. S3b, see online supplementary material). This expression pattern suggests that the consistently infection-induced expression of *PbrChiA* in R-532 may be associated with its resistant phenotype. Further sequence analyses revealed that the CDS and promoter sequences of *PbrChiA* displayed 97.3% and 82.5% similarity between the two varieties, respectively (Fig. S4, see online supplementary material). Additionally, *cis*-acting elements analysis showed a similar element composition in both varieties (Table S6, see online supplementary material), suggesting that the infection-induced differential expression of *PbrChiA* may be attributed to the variations in their upstream transcriptional regulatory networks. To explore the role of *PbrChiA* in pear disease resistance, we transiently silenced *PbrChiA* in R-532 leaves and fruits and transiently overexpressed it in S-589, followed by *B. dothidea* infection (Figs S5 and S6, see online supplementary material). Our findings reveal that silencing *PbrChiA* considerably weakened the resistance of R-532. Although transient overexpression enhanced the resistance of S-589 in both leaves and fruits, it could not fully rectify the resistance deficiency in S-589 leaves (Fig. S5, see online supplementary material). This data indicated that PbrChiA plays a positive role in enhancing disease resistance in pear, and it also highlights the resistance disparities between leaves and fruits. Then the qRT-PCR analysis of 12 randomly selected DEGs showed highly consistent expression patterns with TPM values, providing additional support for the reliability of RNA-Seq analysis (Fig. S7, see online supplementary material).

### Functional characterization of PbrChiA

**Figure 4:**
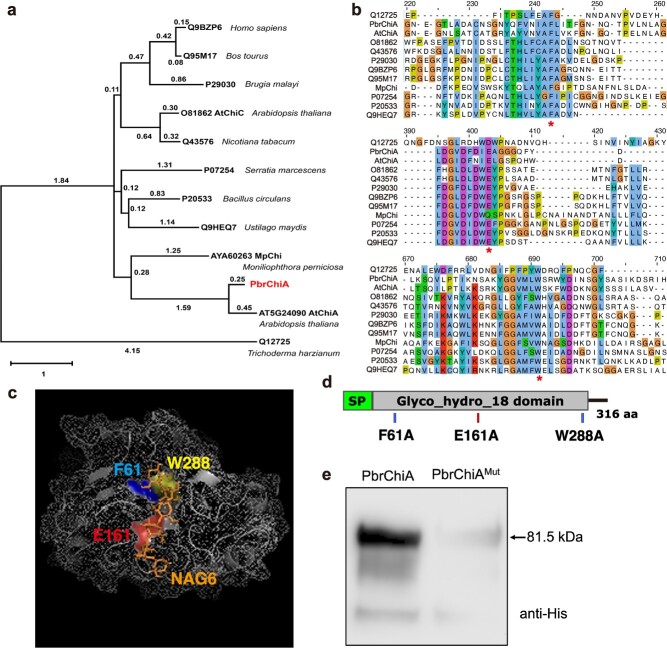
*PbrChiA* encodes a chitinase with chitin-binding ability. (**a**) Maximum likelihood phylogenetic tree of PbrChiA and other tested chitinases. (**b**) Multiple sequence alignment of PbrChiA and other seven GH18 chitinases. Three residues framed by asterisk (F61, E161, and W288) are highly conserved residues in GH18 chitinases for chitin-binding (F61 and W288) and enzyme activity (E161 was predicted to be the catalytic residue of pattern DxxDxDxE). (**c**) Homology modeling of the PbrChiA structure. The 3D model revealed that the three conserved residues are in the catalytic pocket surface with a NAG6. (**d**) Model diagram of artificial substitutions on three conserved residues of PbrChiA^Mut^ protein. SP: signal peptide. Glyco_hydro_18 domain: glycoside hydrolase family 18 conserved domain. (**e**) The chitin beads-based pull-down analysis of PbrChiA and PbrChiA^Mut^ proteins.

The results of the multiple sequence alignment and phylogenetic analysis indicate that PbrChiA is more closely related to *Arabidopsis* Class III chitinase A (AtChiA, AT5G24090) and contains predicted amino acid residues for chitin binding (F61 and W288) and conserved catalytic activity (E161 of DxxDxDxE pattern) (Fig. 4a and b). Furthermore, homology modeling of PbrChiA revealed the three conserved substituted residues (F61, E161, and W288) formed an interface in the catalytic pocket (Fig. 4c). Then, subcellular localization experiments demonstrated that the GFP fluorescence of *PbrChiA-GFP* and *PbrChiA^Mut^-GFP* (which contains three artificial mutations at above conserved sites of PbrChiA as shown in Fig. 4d and Fig. S8b, see online supplementary material) were observed in the intercellular space (Fig. S9a, see online supplementary material), indicating that PbrChiA is a secretory protein and the mutations did not affect its subcellular localization.

To test the functional activity of PbrChiA, purified PbrChiA and PbrChiA^Mut^ proteins were used to perform chitin-beads pull-down and anti-fungal assays with *B. dothidea* scraped mycelium. The results showed that the multiple mutation virtually abolished the chitin binding ability of PbrChiA^Mut^ protein (Fig. 4e), and only PbrChiA protein could break the mycelium of *B. dothidea in vitro* (Fig. S9b, see online supplementary material). Taken together, these results provide evidence that PbrChiA can directly exert antifungal functions as a secretory chitinase that is dependent on its chitin-binding ability to break fungal mycelium, which is consistent with the previous results (Figs S5 and S6, see online supplementary material).

### Ectopic overexpression of *PbrChiA* in *Arabidopsis* increased resistance to *B. dothidea*

**Figure 5:**
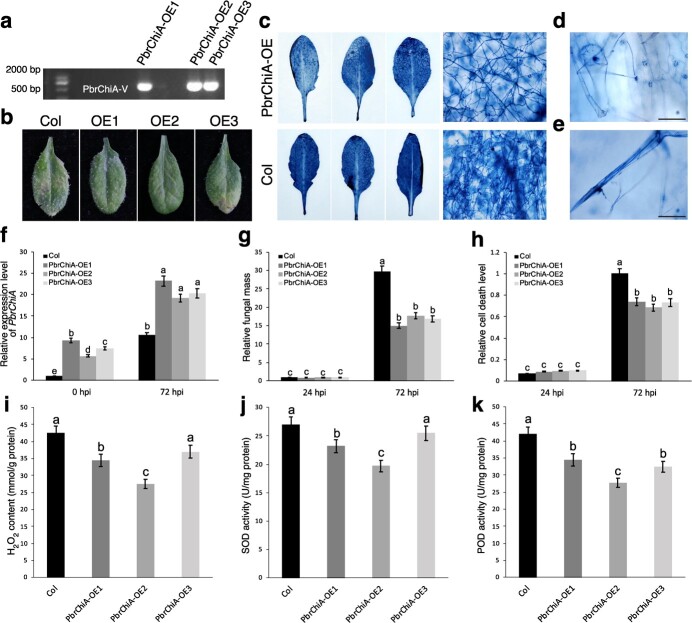
Ectopic expression of *PbrChiA* in *Arabidopsis* increased resistance to *Botryosphaeria dothidea*. (**a**) Identification of the transgenic *Arabidopsis* at DNA level with PCR assays. (**b**) Phenotypes of Col, *PbrChiA-OE1*, *OE2*, and *OE3* infected with *B. dothidea* for 3 days. (**c**) Trypan blue staining for cell death and mycelia in the leaves of the Col and *PbrChiA-OE* transgenic *Arabidopsis* infected with *B. dothidea* for 3 days. (**d**–**e**) Stomata (**d**) and epidermal hairs (**e**) of Col leaves infected by *B. dothidea* mycelia. Bar = 50 μm. (**f**) Relative expression level of *PbrChiA* in Col and *PbrChiA-OE* transgenic *Arabidopsis*. (**g**) Relative fungal mass measured by qRT-PCR. (**h**) Relative cell death measured by ImageJ. (**i**) Measurement of H_2_O_2_ content. (**j**–**k**) Measurement of SOD (**j**) and POD (**k**) activities. Bars with different letters are significantly different at *P* < 0.05 according to Tukey’s single-factor test. Data are shown as mean ± SD. At least three biological repetitions were performed.

To investigate the function of PbrChiA *in vivo*, the 35S promoter-driven overexpression vector was introduced into wild-type *Arabidopsis thaliana* ‘Columbia’ using an *Agrobacterium*-mediated method. The PbrChiA-OE transgenic *A. thaliana* was identified by PCR and qRT-PCR (Fig. 5a and f). The transgenic *A. thaliana* leaves were infected with *B. dothidea* for three days. Ectopic expression of *PbrChiA* in *A. thaliana* increased resistance to *B. dothidea* infection in OE lines, resulting in less fungal biomass and cell death (Fig. 5b, c, g, and h). In addition, the higher expressions of defense related genes were detected in infected leaves of OE lines, including SA-related *PR1* and *PR5* and JA-related *PR3* (Fig. S10a, see online supplementary material). Microscopic observation revealed that *B. dothidea* infection in *A. thaliana* leaves mainly occurred in stomata and epidermis (Fig. 5d and e). H_2_O_2_, one of the important forms of ROS, and ROS-related enzyme activities mark the activation of plant immune response [16]. Interestingly, in this study, ROS accumulation and the enzyme activities of SOD and POD were highest in the WT (Col), while ectopically expressing *Arabidopsis* displayed decreased ROS, indicating that PbrChiA suppressed the immune response level of *Arabidopsis* to *B. dothidea* inoculation (Fig. 5i–k), which is inconsistent with enhanced resistance of OE lines.

### PbrChiA interact with PbrLYK1b2

**Figure 6:**
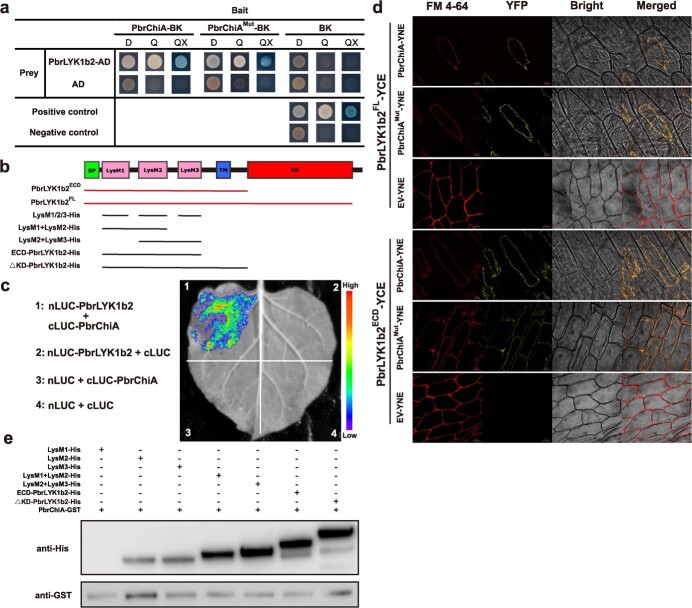
PbrChiA interacts with PbrLYK1b2. (**a**) Point-to-point Y2H verification. The PbrChiA and PbrChiA^Mut^ all interact with PbrLYK1b2. D: SD/−trp/−leu medium. Q: SD/−trp/−leu/−his/−ade medium. QX: SD/−trp/−leu/−his/−ade medium with x-gal. (**b**) Model diagram of PbrLYK1b2 protein. SP: signal peptide. LysM: lysin motif. TM: transmembrane domain. KD: kinase domain. The two uper lines indicate the sequences for BiFC analysis, and the other lines indicate the sequences for GST-based pull-down analysis. (**c**) Luciferase complementation imaging analysis between PbrChiA and PbrLYK1b2. Fluorescence on the leaf surface indicates protein–protein interaction. (**d**) Bimolecular fluorescence complementation analysis between PbrChiA and PbrLYK1b2. The full-length and segmental PbrLYK1b2 all interact with natural or mutated PbrChiA *in vivo*. Bar = 20 μm. (**e**) GST-based pull-down analysis between PbrChiA and PbrLYK1b2. PbrChiA interacts with LysM2 and LysM3 of PbrLYK1b2.

Given the lower levels of ROS and related enzyme activities in PbrChiA-OE *Arabidopsis*, we hypothesized that PbrChiA, which functions as an anti-fungal protein in pear defense against *B. dothidea*, may also suppress ROS accumulation through interactions with other proteins. To investigate this possibility, we performed a yeast two-hybrid screen using a cDNA library obtained from *B. dothidea*-infected pear leaves. We obtained 30 positive clones (Table S7, see online supplementary material), ten of which are associated with plant immunity, including a lysin-motif-containing receptor-like protein, PbrLYK1b2. Previous studies have shown that *PbrLYK1b2* is homologous to *AtCERK1* and induced by *B. dothidea* infection, making it a potential membrane-localized PRR [17], which is consistent with our observation that *PbrLYK1b2*’s expression consistently declined in both R-532 and S-589 in later infection stages (Fig. S10b, see online supplementary materia). Furthermore, an endo-chitinase from non-infected total protein of apple can be pulled down by GST-MdCERK1 *in vivo* [18], but the biological implications of this interaction remain to be elucidated. To confirm whether PbrChiA interacts with PbrLYK1b2, we utilized point-to-point Y2H verification, luciferase complementation imaging assay (LCI), bimolecular fluorescence complementation (BiFC) assay, and *in vitro* GST-based pull-down assay. Our point-to-point Y2H results confirmed that full-length PbrLYK1b2 can interact with both PbrChiA and PbrChiA^Mut^ (Fig. 6a). The fluorescence on the leaf surface also indicated protein–protein interaction between them (Fig. 6c). Further verification was provided by the BiFC and pull-down assays, which demonstrated that the interaction between PbrChiA (or its mutant form) and the extracellular domains of PbrLYK1b2 occurred (Fig. 6d and e). Co-expression of *PbrChiA* or *PbrChiA^Mut^* with *PbrLYK1b2-FL* or *PbrLYK1b2-ECD* in onion epidermal cells presented YFP fluorescence, confirming PbrLYK1b2 as the genuine interacting protein of PbrChiA and indicating the mutations did not affect the interaction (Fig. 6d). Additionally, the GST-based pull-down results indicated that the interaction mainly occurs in LysM2 and LysM3 of PbrLYK1b2 (Fig. 6b and e).

### PbrChiA and PbrLYK1b2 positively regulate pear resistance to *B. dothidea* infection

**Figure 7:**
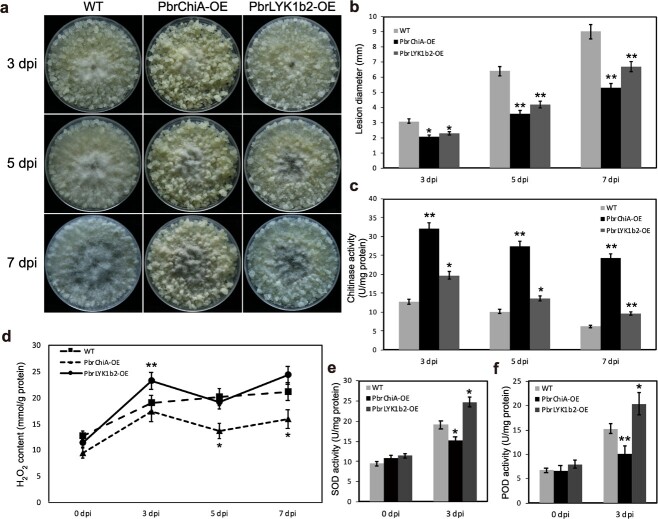
Over-expressing of *PbrChiA* or *PbrLYK1b2* conferred enhanced resistance to *B. dothidea* infection in pear callus. (**a**) Phenotypes of WT and transgenic pear callus infected by *B. dothidea*. (**b**) Measurement of the lesion diameters of WT and transgenic pear callus. (**c**) Measurement of chitinase activities of WT and transgenic pear callus. (**d**) Measurement of H_2_O_2_ content of WT and transgenic pear callus. (**e**–**f**) Measurement of SOD (**e**) and POD (**f**) activities of WT and transgenic pear callus. Data are shown as mean ± SD. At least three biological repetitions were performed. The data were analysed using two-way ANOVA followed by Sidak’s test. ^**^P < 0.01, ^*^P < 0.05.

To investigate the potential roles of PbrChiA and PbrLYK1b2 in pear’s defense against *B. dothidea in vivo*, stable overexpression cell lines of *PbrChiA* (PbrChiA-OE) and *PbrLYK1b2* (PbrLYK1b2-OE) were generated in pear callus, respectively (Fig. S11a and c, see online supplementary materia). Resistance to *B. dothidea* infection was then tested in WT and the two types of OE callus (Fig. 7).

Although no significant differences in phenotype were observed between the WT and the two types of OE callus without infection (Fig. S12, see online supplementary materia), significant differences in fungal growth were observed upon inoculation with *B. dothidea*. Both types of OE callus demonstrated greater resistance against *B. dothidea* infection, with significantly smaller mycelial diameter, slower mycelial growth and higher expressions of defense related genes compared to WT callus (Fig. 7a and b; Fig. S10c, see online supplementary materia), but exhibited different patterns of change in H_2_O_2_ content and the activities of chitinase, SOD, and POD (Fig. 7c–f). The total chitinase activity of both OE callus was higher compared to WT, while that of PbrChiA-OE was the highest (Fig. 7c). Despite an increased activity of SOD and POD after infection, their activities were lower in PbrChiA-OE callus compared to WT (Fig. 7e and f), which is consistent with the results in PbrChiA-OE transgenic *Arabidopsis*. As for PbrLYK1b2-OE callus, the H_2_O_2_ content and related enzyme activity were all significantly higher than WT (Fig. 7d–f). Additionally, the overexpression of *PbrChiA* also enhanced the resistance of OE callus to other fungal diseases *Alternaria alternata* H1 and *Colletotrichum fructicola* WD40 (Fig. S13, see online supplementary material), suggesting *PbrChiA* is associated with the broad-spectrum resistance to fungal disease.

**Figure 8:**
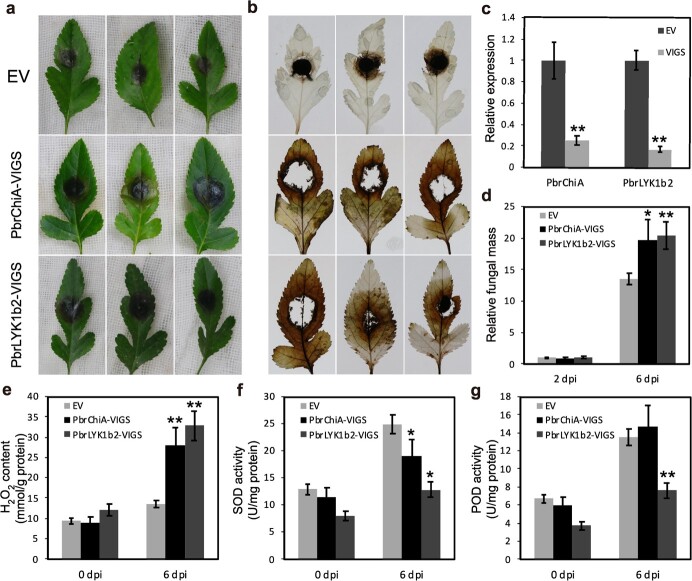
Gene silencing on *PbrChiA* or *PbrLYK1b2* reduced the resistance to *Botryosphaeria dothidea* infection in *Pyrus betulaefolia* leaves. **(a**–**b**) Phenotypes (**a**) and DAB (**b**) staining of *Pyrus betulaefolia* seedling leaves infected by *B. dothidea*. EV: leaves treated with the *Agrobacterium tumefaciens* GV3101 strain containing *pTRV1* and *pTRV2* vector plasmid. PbrChiA-VIGS: leaves treated with the GV3101 strain containing *pTRV1* and *pTRV2-PbrChiA*. PbrLYK1b2-VIGS: leaves treated with the GV3101 strain containing *pTRV1* and *pTRV2-PbrLYK1b2*. (**c**) Relative expression of *PbrChiA* or *PbrLYK1b2* in pear seedling leaves infected by *B. dothidea*. The expression of *PbrChiA* or *PbrLYK1b2* were significantly silenced. (**d**) Relative fungal mass of infected PbrChiA-VIGS and PbrLYK1b2-VIGS leaves. The fungal biomass of infected pear seedling leaves was determined at 2 and 6 dpi by qRT-PCR using specific primers for the *BdActin1* gene of *B. dothidea* and normalized to the *PbrTUB* gene. (**e**) Measurement of H_2_O_2_ content of pear seedling leaves infected by *B. dothidea*. (**f**–**g**) Measurement of SOD (**f**) and POD (**g**) activities of pear seedling leaves infected by *B. dothidea*. The data were analysed using two-way ANOVA followed by Sidak’s test. ^**^P < 0.01, ^*^P < 0.05.

On the other hand, when *PbrChiA* or *PbrLYK1b2* expression was silenced using VIGS, larger lesions, higher fungal mass, higher H_2_O_2_ content and lower SOD activity were observed on leaves following *B. dothidea* infection (Fig. 8a–f). Silencing *PbrLYK1b2* also down-regulated the POD activity (Fig. 8g) and the expression of *PR1* and *PR5* (Fig. S10d, see online supplementary material). In addition, ectopic overexpression of *PbrLYK1b2-KD* induced cell death in *Nicotiana benthamiana* leaves (Fig. S11b, see online supplementary material). These results suggest that PbrLYK1b2 positively regulates pear resistance to *B. dothidea* infection by upregulating the ROS-related immune response, and PbrChiA is also a positive regulator of pear resistance as a secreted anti-fungal chitinase and suppresses infection-induced ROS accumulation.

### The suppression on chitin-induced ROS accumulation from PbrChiA depend on PbrLYK1b2

**Figure 9:**
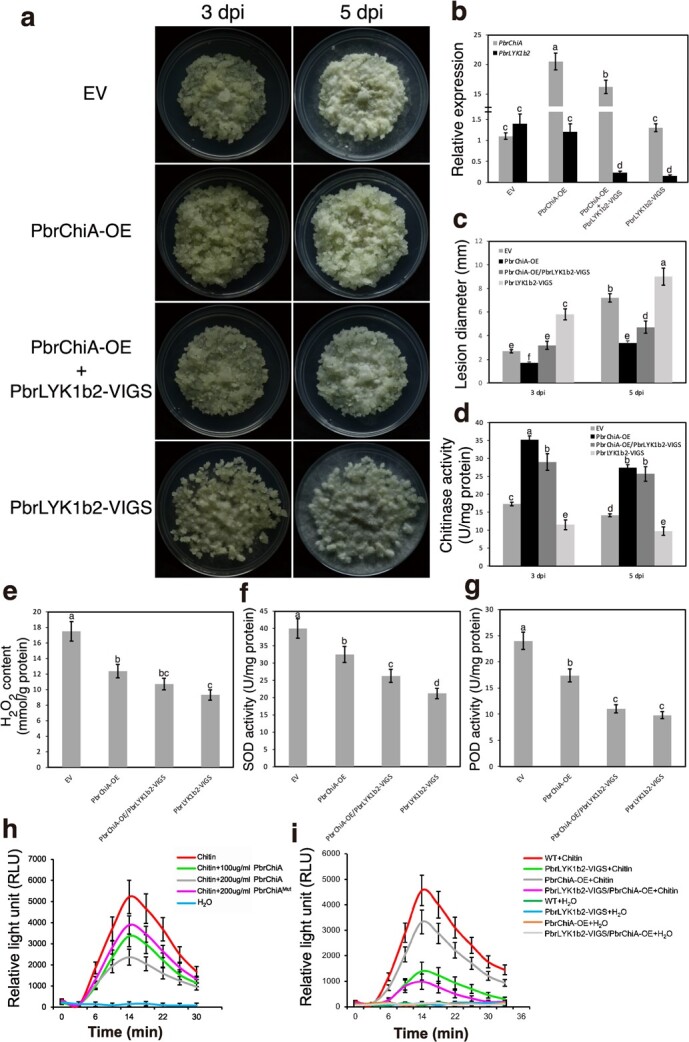
The suppression on chitin-induced ROS accumulation from PbrChiA depend on PbrLYK1b2. (**a**) Phenotypes of WT and transgenic pear callus infected by *B. dothidea*. EV: pear callus treated with the *Agrobacterium tumefaciens* GV3101 strain containing *pTRV1* and *pTRV2* vector plasmid. PbrLYK1b2-VIGS: callus treated with the GV3101 strain containing *pTRV1* and *pTRV2- PbrLYK1b2*. PbrChiA-OE/PbrLYK1b2-VIGS: PbrChiA-OE callus treated with the GV3101 strain containing *pTRV1* and *pTRV2- PbrLYK1b2*. (**b**) Relative expression of *PbrChiA* or *PbrLYK1b2* in pear callus infected by *Botryosphaeria dothidea*. The expression of *PbrChiA* or *PbrLYK1b2* were significantly silenced. (**c**) Measurement of the lesion diameters of pear callus infected by *B. dothidea*. (**d**) Measurement of chitinase activities of pear callus infected by *B. dothidea*. (**e**) Measurement of H_2_O_2_ content of pear callus infected by *B. dothidea*. (**f**–**g**) Measurement of SOD (**f**) and POD (**g**) activities of pear callus infected by *B. dothidea*. (**h**) Measurement of H_2_O_2_ generation in pear callus in response to chitin treatment. The WT pear callus pretreated with H_2_O, 100 μg/ml PbrChiA, 200 μg/ml PbrChiA and 200 μg/ml PbrChiA^Mut^, respectively, were then treated with 50 μg/mL chitin mixture. The H_2_O treated WT callus were as control. (**i**) Measurement of H_2_O_2_ generation in pear callus in response to chitin treatment. After 12 h recovery, the EV, PbrChiA-OE, PbrLYK1b2-VIGS, and PbrChiA-OE/PbrLYK1b2-VIGS pear callus were treated with 50 μg/mL chitin mixture. The H_2_O treated seedlings were as control. Bars with different letters are significantly different at *P* < 0.05 according to Tukey’s single-factor test. Data are shown as mean ± SD. At least three biological repetitions were performed.

In our previous results, the resistant variety R-532 displayed a decreasing trend in ROS levels and related enzyme activities at later stages of infection, similar to the phenotype observed in PbrChiA-OE *Arabidopsis* and pear callus, and the PbrChiA-interacting protein PbrLYK1b2 positive regulates the ROS-related immune response. These results indicated that high-level expression of PbrChiA may suppress ROS accumulation by interacting with PbrLYK1b2 and be closely associated with enhanced resistance. To test this hypothesis, we silenced *PbrLYK1b2* expression in WT and PbrChiA-OE pear callus, which were then treated with *B. dothidea* (Fig. 9a–g). Following *B. dothidea* infection, PbrLYK1b2-VIGS/PbrChiA-OE callus showed greater susceptibility than PbrChiA-OE, meaning the enhanced resistance of PbrChiA-OE pear callus partly relay on PbrLYK1b2.

Furthermore, as showed in Fig. 9h, the chitin-induced ROS in WT callus was reduced by treatment with either PbrChiA or PbrChiA^Mut^ protein. As the concentration of PbrChiA protein increased, the treated groups showed a downward trend in ROS peak level. These findings indicate that PbrChiA protein can suppress chitin-induced ROS in pear callus, and this effect is partial dependent on chitinase activity (Fig. 9h). In addition, with chitin treatment, PbrChiA-OE callus exhibited lower ROS levels than WT but higher levels than PbrLYK1b2-VIGS (Fig. 9i). However, the overexpression of *PbrChiA* did not significantly reduce chitin-induced ROS accumulation in PbrLYK1b2-VIGS/PbrChiA-OE callus, suggesting that the ROS suppression from PbrChiA depends, to some extent, on its interaction with PbrLYK1b2.

## Discussion

Although chitinase have been implicated in the immune defense of various plants, it remains unclear whether they play a similar role and exhibit specific mechanisms in the fungal immune response of pear trees. In this study, we identified a GH18 family chitinase, PbrChiA, through comparative transcriptome analysis and genetic transformation-based functional verification. We partially elucidated its mechanism on the resistance of pear to *B. dothidea*. Specifically, PbrChiA acts as a secreted chitinase that directly exerts anti-fungal function while also inhibiting chitin-induced ROS accumulation by interacting with PbrLYK1b2.

We obtained resistant pear variety R-532 and susceptible variety S-589 through preliminary resistance identification against *B. dothidea*. After inoculation with *B. dothidea*, both genotypes exhibited similar infection-induced defense responses in the early infection stage, including a decrease in PAL enzyme activity, a significant increase in ROS levels and ROS-related enzyme activity (Fig. 1), and reprogramming of gene expression in similar KEGG pathways (Fig. 2). Phenylalanine ammonia-lyase (PAL) is an enzyme closely related to the SA biosynthesis pathway, which plays a critical role in plant defense against biotic stress [19, 20]. In *Populus tomentosa*, large amounts of SA accumulated after inoculation with *B. dothidea* [21]. Under such conditions, plants accumulate endogenous SA to accumulate ROS and initiate the expression of defense-related genes, thereby activating systemic acquired resistance (SAR) [22–24]. Our findings suggest that *B. dothidea* infection activates stronger defense responses in R-532 at early stage. However, after 48 hpi, obvious lesions appeared and gradually expanded on the leaves of both R-532 and S-589, indicating that early defense responses failed to restrict *B. dothidea* infection. Therefore, the faster lesion expansion indicated that different resistance phenotypes between R-532 and S-589 may originate from the later necrotrophic infection stage of *B. dothidea* after successful colonization.

In the later infection stages (from 48 hpi to 144 hpi), PAL enzyme activity in R-532 remained consistently high after reaching its peak. This suggests that R-532 might accumulate a greater amount of antimicrobial substances. Notably, in the resistant variety R-532, although the activity of ROS-related enzymes remained higher than in S-589 under a continuous declining trend, it accumulated less ROS, possibly due to certain inhibition mechanisms of *B. dothidea*. While ROS-induced HR is known to be unfavorable for *B. dothidea* infection at early stage, ROS-induced cell death in the later stages is beneficial for the pathogen’s acquisition of nutrients and continued expansion [14, 15]. In other words, inhibiting ROS during the later necrotrophic stage may be disadvantageous for necrotrophic pathogenic fungi *B. dothidea*. Thus, this plant-beneficial ROS suppression effect may be driven by the plants themselves to slow down *B. dothidea* expansion. However, it remains unclear how pears balance their ROS-mediated immune responses and the susceptibility caused by high-level ROS accumulation during infection.

Then, through comparative transcriptome analysis, we identified a GH18 chitinase-encoding gene, *PbrChiA*, which was specifically highly expressed in the later infection stages on resistant variety R-532 (Fig. S3, see online supplementary material). Chitinase, a vital component of PR proteins, often shows a positive correlation between its abundance and plant resistance [25, 26]. Our transient transgenic analyses of *PbrChiA* in pear leaves and fruits reaffirm this relationship. The results indicated the crucial role PbrChiA plays in bolstering the greater resistance of R-532, as observed in Figs S5 and S6 (see online supplementary material). Notably, *Pbr034281.1* and *Pbr034283.5* genes also showed the similar expression pattern to *PbrChiA* which suggested that they might provide valuable insights into the overall understanding of the GH18 chitinase involved resistance system in pear (Fig. S3, see online supplementary material).

As Fiorin *et al.* reported, the neofunctionalization of secreted enzymes is one of the conserved pathways of fungal effector evolution. Some pathogens release inactivated chitinases (like MpChi) during the early stage of infection, which affects the activation of plant immunity by binding to free chitin fragments [2]. Similarly, in the resistant variety R-532, there are specific lower ROS accumulation and high-level expression of *PbrChiA* during the later stage, and thus, it was hypothesized that PbrChiA might be involved in the immune inhibition. Currently, there is little to report on the neofunctionalization in plant GH18 chitinase. However, in the field of evolutionary biology, it is known that the function and structure of proteins evolve and change over time. Therefore, it is necessary to further investigate the GH18 chitinase family to determine whether such enzymes have undergone similar evolution as MpChi. Such research will contribute to a better understanding of the evolutionary history of chitin-related pathways in plants and provide a stronger theoretical basis for related applications. By overexpressing *PbrChiA*, it is possible to increase the level of chitinase activity in plants, thereby enhancing its ability to break down fungal cell walls and resist infection. Despite that, it is important to note that overexpression of any gene can have unintended consequences, such as affecting the plant’s growth or other metabolic pathways. Although the *PbrChiA* overexpression conferred enhanced broad-spectrum fungal disease resistance on pear callus (Fig. 7; Fig. S13, see online supplementary material), further research and testing would also be necessary to determine the efficacy and safety of using *PbrChiA* overexpression as a strategy for enhancing plant resistance to fungal infections.

To test the hypothesis about PbrChiA inhibiting immune response, various *in vitro* and *in vivo* analyses were performed to systematically study the biological functions of PbrChiA. Chitin-binding analysis, anti-fungal experiments, and subcellular localization analysis results showed that PbrChiA is a secreted anti-fungal chitinase that can directly break *B. dothidea* mycelium *in vitro* through its chitinase activity (Fig. 4e; Fig. S7, see online supplementary material). Although it is known that pear is a difficult experimental system to obtain transgenic regenerated plants, the stable genetic transformation system of susceptible cultivar ‘Qieli’ (*Pyrus communis* L.) callus and the transient silencing system of resistant variety ‘Duli’ (*Pyrus betulifolia* B.) have become relatively mature and widely applied in the investigation of gene function [27–32]. As expected, overexpression of *PbrChiA* in *Arabidopsis* and pear callus enhanced its disease resistance might by increasing chitinase activity and the expression of defense-related genes (Figs 5 and 7; Fig. S10a, see online supplementary material). Surprisingly, similar to what was observed in R-532, PbrChiA-OE *Arabidopsis* and pear callus lines showed lower ROS-related enzyme activities and accumulated less ROS when challenged with *B. dothidea* infection. Moreover, purified PbrChiA protein and mutant protein PbrChiA^Mut^ applied exogenously could inhibit chitin-induced ROS accumulation in WT callus (Fig. 9h). Overall, these results demonstrate that the accumulation of PbrChiA can directly participate in inhibiting chitin-induced ROS accumulation, and this inhibitory effect on immune responses may be different from the reported inactivated chitinase effector MpChi.

To further elucidate the mechanism by which PbrChiA inhibits ROS, an interacting protein of PbrChiA, PbrLYK1b2, was identified (Fig. 6). Previous studies have shown that *PbrLYK1b2* is one of the homologs of *AtCERK1* in pear and is induced by *B. dothidea* infection [33]. The function of AtCERK1 in mediating chitin signaling pathways has been widely reported, including the activation of downstream MAPK cascades, activation of defense-related gene expression, and ROS burst [6–9]. Additionally, in *Arabidopsis* and rice, the LysM2 motif in the extracellular domain of CERK1 has been reported as the main structure for recognizing and binding chitin oligomer fragments [34, 35]. Therefore, it is speculated that the interaction between PbrChiA and PbrLYK1b2 may be the basis for their ROS-inhibiting effect. Then, the biological function of PbrLYK1b2 was explored, and the segment of their interaction was further verified. Our results demonstrated that PbrLYK1b2 can enhance the disease resistance by positively regulating ROS-related responses and the expression of *PR1* an *PR5* in a SA-related pathway (Figs 7 and 8; Fig. S10c, see online supplementary material), and that PbrChiA can bind to the LysM2 and LysM3 motifs in the extracellular domain of PbrLYK1b2 (Fig. 6d). Furthermore, the inhibitory effect of PbrChiA on ROS-related responses is partially dependent on PbrLYK1b2 (Fig. 9). Based on these findings, we hypothesize that the interaction with PbrChiA partially blocks the chitin binding of PbrLYK1b2, affecting chitin signaling and inhibiting ROS accumulation. However, an apparent inconsistency arises between the results of H_2_O_2_ levels presented in Figs 8e and 9e. One possible explanation is that the impaired activation of immune responses in PbrLYK1b2-VIGS leaves might facilitate a more extensive and severe *B. dothidea* progression by 6 dpi, culminating in heightened ROS accumulation (Fig. 8e). On the other hand, the changes detected in callus mainly reflect the localized alterations in the infected region. Consequently, the ineffectively activated ROS bursts in PbrLYK1b2-VIGS callus at 3 dpi would lead to lower H_2_O_2_ levels compared to EV (Fig. 9e). This theory aligns with the H_2_O_2_ levels observed in the PbrLYK1b2-OE callus at 3 dpi, as illustrated in Fig. 7d. In addition, this could also be due to differences between the defense responses induced by *B. dothidea* in leaves and callus cells, which is consistent with the results of transient transgenic analyses of *PbrChiA* (Figs S5 and S6, see online supplementary material). At present, no specific amino acid residue has been identified in either PbrChiA or PbrLYK1b2 that interacts with each other. Therefore, further analysis through artificial mutation is needed to elucidate the inhibition mechanism of PbrChiA on ROS.

**Figure 10:**
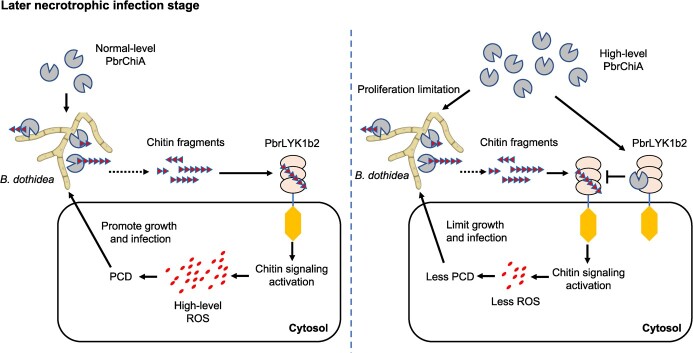
Graphical abstract. The induced high-level expression of *PbrChiA* can positively regulate pear resistance to *Botryosphaeria dothidea*, which is dependent not only on the anti-fungal activity from PbrChiA but also on the interaction with PbrLYK1b2 to appropriately suppress chitin-induced ROS accumulation. The higher antifungal activity and the lower ROS accumulation at later necrotrophic stages combined to confer the resistance against *B. dothidea*.

In conclusion, the induction of high-level expression of *PbrChiA* positively regulates pear resistance to *B. dothidea* (Fig. 10). This process relies not only on the anti-fungal activity of PbrChiA but also on the interaction with PbrLYK1b2, which appropriately suppresses chitin-induced ROS accumulation. These findings suggest that the combination of higher anti-fungal activity and lower ROS accumulation at later necrotrophic infection stages may be an effective strategy for enhancing plant resistance to fungal infections. This model provides valuable insights into the molecular mechanisms involved in the plant-fungal interaction, specifically the role of chitinases in enhancing plant resistance to *B. dothidea* infection. Moreover, in the future, it would be interesting to investigate whether plant GH18 chitinases have undergone neofunctionalization similar to MpChi and other fungal effectors. Such research could contribute to a better understanding of the evolutionary history of chitin-related pathways in plants and provide a stronger theoretical basis for related applications.

## Materials and methods

### Plant materials and pathogen inoculation

The resistant variety R-532 (*Pyrus bretshneider* Rehd, ‘Jingxing’) and susceptible variety S-589 (*Pyrus bretshneider* Rehd, ‘Jixue’) were obtained from the pear germplasm orchard of the Center of Pear Engineering Technology Research situated at Hushu in Nanjing of Nanjing Agricultural University. *B. dothidea* LW-54 and anthracnose pathogen *C. fructicola* WD40 strains were donated by the laboratory of Fengquan Liu, Institute of Plant Protection, Jiangsu Academy of Agricultural Sciences (Nanjing, China). The black spot pathogen *A. alternata* H1 strains were isolated and donated by Qiao Qinghai, a doctoral student in our laboratory. The fourth and fifth leaves of each variety were detached from two-year-old mature shoots and allowed to recover for 12 hours before infection.

The *B. dothidea* mycelia infection treatment followed a previously reported method [17]. The control and infected leaves were cultured at 25°C in darkness for 6 days. Leaves were sampled at 0 hpi (hour post-infection), 12 hpi, 48 hpi, 96 hpi, and 144 hpi for lesion diameter measurement, physiological indicator determination, and RNA sequencing with three replicates for each. For the leaves with no obvious lesion at 0 hpi and 12 hpi, circular leaf discs with a diameter of 1 cm were collected from the inoculation site. For the leaves that showed obvious lesion at 48 hpi, 96 hpi, and 144 hpi, circular leaf discs were taken at the border between the diseased area and the unaffected area, with the proportion of the diseased area being approximately one-third of the total disc.

The leaves of wild-type and transgenic *A. thaliana* were detached from the same growth state, and then were placed upside down on a PDA medium containing *B. dothidea* mycelia for three days. The leaves were kept in humidity conditions at 25°C under a 16-h/8-h light/dark period. About 15 leaves of each line were inoculated each time.

H_2_O_2_ contents and the activities of chitinase, PAL, SOD, and POD were measured using the test kits (Nanjing Jiancheng Bioengineering Institute, Nanjing, China). The fungal biomass was determined by qRT-PCR using specific primers for the *BdActin* gene of *B. dothidea*, and normalized to the *PbrTUB* or *AtActin* genes according to previously reported methods [33, 36]. Three biological repetitions were performed with about 15 leaves of each.

### RNA sequencing and differential expression analysis

The total RNA was extracted by using extraction kit (Tiangen, Beijing, China). All 30 libraries were constructed and sequenced using the Illumina platform at Majorbio Co. Ltd (Shanghai, China). The raw paired end reads were trimmed and quality controlled by fastp (https://github.com/OpenGene/fastp) with default parameters. Then clean reads were aligned to reference genome (Chinese white pear, *Pyrus bretshneider* Rehd, ‘Dangshansuli’, v1.0) with orientation mode using HISAT2 (http://ccb.jhu.edu/software/hisat2/index.shtml) software. The mapped reads of each sample were assembled by StringTie (https://ccb.jhu.edu/software/stringtie/) in a reference-based approach. The TPM values of gene expression level were calculated which were subsequently used for further analysis (Table S8, see online supplementary material).

DEG (differentially expressed gene) analysis was performed by R::DESeq2 package v1.10.1 based on read counts. The DEGs from the early infection stage were obtained from the comparisons between 0 hpi and 12 hpi samples. Meanwhile, the DEGs from each later infection stage were identified through the comparisons between the samples from R-532 and S-589. DEG was assigned as the gene with an adjusted *P*-value of <0.001, absolute fold change of ≥1.8, and a TPM of >1 in at least one sample. The temporal differential expression analysis was performed using the R::maSigPro package with default parameters, and S-589 was as the control group. Gene Set Enrichment Analysis (GSEA) and Kyoto Encyclopedia of Genes and Genomes (KEGG) pathway enrichment analyses were all performed by online functions provided by Majorbio.

### Identification of Glycosyl hydrolases 18 genes in Chinese white pear

The seed file of Glycosyl hydrolases 18 family (PF00704) was used to perform HMM-based (Hidden Markov Model) search against the protein database of the Chinese white pear genome by using HMMER3 software with E-values <1e^−10^. The NCBI Batch CD-Search tools (Batch CD-Search: https://www.ncbi.nlm.nih.gov/Structure/bwrpsb/bwrpsb.cgi) based on CDD v3.18 was used to verify the existence of the GH18 domain with E-values <1e^−6^ (Table S5, see online supplementary material). The multiple sequence alignment and maximum-likelihood phylogenetic tree of PbrChiA and other 11 reported Glycosyl hydrolases 18 chitinases were performed by Clustal Omega online tool (https://www.ebi.ac.uk/Tools/msa/clustalo/) (Fig. S8a and Table S9, see online supplementary material).

### Structural modeling

Homology modeling of signal peptide removed PbrChiA (residues 36–316) was performed by using the Phyre2 online tool with normal modeling mode [37]. The final model presented a confidence score of 100% and a coverage of 93% and was based on a GH18 chitinase from *Punica granatum* (Protein Data Bank ID 4TOQ, 100% confidence and 65% identity). Prediction of binding sites was performed with 3DLigandSite [38] and matched previous experimental evidence for GH18 proteins [39–43]. Molecular docking was performed on the SwissDock server [44] with NAG6 as the ligand. Visualization and edition of the model was done with PyMol v2.5.4.

### Subcellular localization of PbrChiA

The subcellular localization assay was performed as previously described [17]. Briefly, the *Agrobacterium tumefaciens* containing *p1300-target* was transiently expressed in *Nicotiana benthamiana* leaves. After two days, the GFP signals were detected under 2 M NaCl solution using confocal laser scanning microscope (Zeiss LSM 780, Germany). The empty vector *pCAMBIA1300-GFP* was used as the control.

### Stable transformation on *Arabidopsis*

To investigate the function of target genes, the 35S promoted stable overexpression vectors were introduced into ‘Columbia’ *Arabidopsis* using an *Agrobacterium*-mediated method, but the PbrLYK1b2-OE transgenic *Arabidopsis* was not successfully constructed may for its death-inducing effect. Briefly, the full-length ORF of *PbrChiA* and *PbrLYK1b2* was amplified and subcloned into *pCAMBIA1300-GFP* vector driven by the 35S promoter between the sites *XbalI* and *BamHI* by homologous recombination to generate the overexpression vector *p1300-PbrChiA* and *p1300-PbrLYK1b2*, respectively. The MS medium containing 20 mg/L hygromycin was used to select positive seeds. The T3 transgenic lines were then verified on DNA (by PCR) and RNA (by qRT-PCR) levels. All used primers were listed in Table S10 (see online supplementary material).

### Yeast two-hybrid

For large-scale yeast two-hybrid (Y2H) screening, a normalized *pGADT7*-based library made from *B. dothidea* infected leaves were used. For yeast two-hybrid assay, the *PbrChiA* cDNA bait without signal peptide was cloned into *pGBKT7* between *EcoRI* and *BamHI* resulting in *pGBKT7-PbrChiA*. The fused plasmids were co-transformed into the yeast stain and cultivated on high stringency SD growth medium (−Leu/−Trp/−Ade/–His) supplemented with 40 μg/ml X-α-Gal and 100 ng/ml Aureobasidin A at 30°C. The DNA sequence from blue colonies was extracted and sequenced. The point-to-point Y2H verifications were performed as described above. The full-length and ECD sequences of *PbrLYK1b2* were subcloned into *pGADT7* between *EcoRI* and *BamHI*, respectively. All used primers were listed in Table S10 (see online supplementary material).

### Luciferase complementation imaging assay

A LCI assay was performed as described previously [45]. The full-length coding sequences of *PbrChiA* were cloned into the vector *cLUC*, while the full-length coding sequences of *PbrLYK1b2* were cloned into the vector *nLUC*. Then, the GV3101 strain containing paired constructs of *cLUC* plasmid (empty *cLUC*, and *cLUC-PbrChiA*) and *nLUC* plasmid (empty *nLUC* and *nLUC*-*PbrLYK1b2*) were transiently co-expressed in the *N. benthamiana* leaves through Agrobacteria-mediated co-infiltration. The primers used for LCI assay are listed in Table S10 (see online supplementary material).

### Pull down

The protein pull-down assay was performed as previously described [46], and chitin-beads pull-down was performed as the protocol of Chitin Magnetic Beads (E8036S, New England Biolabs) described. Briefly, the chitin-bead based pull-down were performed by using 50 μl chitin magnetic beads (NEB) and 100 μg/ml PbrChiA or PbrChiA^Mut^ proteins at a 4°C shock incubator for 1 h, respectively. After three washes, the chitin-binding proteins were eluted by boiling with SDS-buffer to perform SDS-PAGE and western blot assays. As for the GST-based pull-down assay, the recombinant GST-labeled PbrChiA protein and each His-labeled proteins were mixed and incubated with GST-beads at a 37°C shock incubator for 2 h. After washing, the binding proteins were eluted by boiling with SDS-buffer to perform SDS-PAGE and western blot assays with anti-GST and anti-His primary antibodies (Shenggong, China). The encoding DNA sequence of mutated version of PbrChiA was generated by commercial company (Shenggong, China). Coding sequences of PbrChiA and PbrChiA^Mut^ without signal peptide were subcloned into *pGEX-4 T* between *BamHI* and *EcoRI* by homologous recombination. The segmental sequences of *PbrLYK1b2* were subcloned into *pCold-His* between *XhoI* and *XbaI* by homologous recombination. All used primers were listed in Table S10 (see online supplementary material).

### Bimolecular fluorescence complementation

The full-length coding sequences of PbrChiA and PbrChiA^Mut^ were amplified and subcloned into *pYNE* vector between *XbaI* and *BamHI*, respectively. The ORF of full-length and extracellular domain of *PbrLYK1b2* were subcloned into *pYCE* vector between *XbaI* and *BamHI*. Then, the GV3101 strain containing *pYNE* plasmid (empty *pYNE*, *pYNE-PbrChiA,* and *pYNE-PbrChiA^Mut^*) and *pYCE* plasmid (empty *pYCE pYCE-PbrLYK1b2-ECD*, and *pYCE-PbrLYK1b2-FL*) was transiently expressed in onion epidermal cells in different combination. After two days, the YFP signals were detected under 1 M NaCl solution using confocal laser scanning microscope (Zeiss LSM 780, Germany). All used primers were listed in Table S10 (see online supplementary material).

### Stable transformation on pear callus

Although it is known that pear is a difficult experimental system to obtain transgenic regenerated plants, the stable genetic transformation system of callus cells has become relatively mature and widely applied in the investigation of gene function [27–30]. In order to analysis the biological functions of interested targets, the stable genetic transformation on callus was performed. The transformation on pear callus of susceptible cultivar ‘Qieli’ (*P. communis* L.) refers to the method previously reported with the selection of MS medium containing 20 mg/L hygromycin [31]. The positive calluses were verified on DNA (by PCR) and RNA (by qRT-PCR) levels. All used primers were listed in Table S10 (see online supplementary material).

### Transient transformation and VIGS treatment

It has been previously documented that the overexpression of *AtCERK1* can induce cell death. To explore the potential effects of heterologous overexpression of *PbrLYK1b2*, we infiltrated 4-week-old *N. benthamiana* leaves with *A. tumefaciens* GV3101 carrying either the *p1300* empty vector plasmid, the *p1300-PbrLYK1b2-FL* plasmid, or the *p1300-PbrLYK1b2-KD* plasmid. Observations were made on the fourth day post-infiltration.

To perform the gene silencing on *PbrChiA* and *PbrLYK1b2*, the 500 bp fragment of each was cloned and fused into the *pTRV2* vector between *EcoRI* and *BamHI*, respectively. VIGS was performed on 4-week-old pear seedling leaves of resistant variety ‘Duli’ (*Pyrus betulifolia* Bunge) according to previous reports with the *A. tumefaciens* GV3101 strain containing *pTRV1* and *pTRV2-target* in a 1:1 ratio [32]. The mixture of *A. tumefaciens* containing *pTRV1* and empty *pTRV2* was used as control. The gene silencing of *PbrLYK1b2* on pear callus was performed as described above for stable transformation. The positive materials were verified by qRT-PCR. All used primers were listed in Table S10 (see online supplementary material). As shown in Fig. S14 (see online supplementary material), there are four homologous genes to *PbrChiA* and two homologous genes to *PbrLYK1b2* in *P. betulifolia* Bunge. Remarkably, these identified homologous genes displayed a significant level of sequence similarity to the 500 bp fragments that were specifically targeted for the VIGS treatment. As a result, there is a high probability that these homologous genes might undergo transient silencing during the VIGS procedures.

To transiently overexpress *PbrChiA* in S-589 leaves and fruits, *A. tumefaciens* GV3101 strain containing *p1300-PbrChiA* plasmid was infiltrated. The inoculation was performed at the infiltration site by attaching with *B. dothidea* mycelia 7 d after infiltration. To perform the gene silencing on *PbrChiA* in R-532 leaves and fruits, *A. tumefaciens* strains containing *pTRV2-PbrChiA* and *pTRV1* were equally mixed and co-infiltrated. The resistance was evaluated by measuring the diameter of lesions.

### Histochemistry staining and ROS burst detection

Following previously described methods, ROS accumulation were visualized by DAB staining [47]. Briefly, leaves were incubated in a 3,3-diaminobenzidine (DAB) solution (1 mg/mL) overnight in the dark. Then, leaves were destained with 80% ethanol and placed in a water bath at 65°C. Trypan blue staining was used to detect mycelial growth and cell death according to previously described methods [33]. ROS burst detection was performed as previously described [48]. Briefly, the 0.1 g WT pear callus were placed in a black 96-well plate and placed in the dark for 12 h in sterile water. After gently removing the water, the mixture of 0.2 μM luminol reagent, 10 μg/mL horseradish peroxidase, 50 μg/mL chitin (Shenggong, China) and/or target purified proteins were put into each well as designed.

### Statistical analyses

One-way ANOVA, Student’s *t*-test and Tukey’s single-factor test were performed using Microsoft Excel software v16.75.2. Significant differences were detected by Student’s *t*-tests. In the figures, the following notations are used: *P < 0.05 and **P < 0.01. In figures, bars with different letters are significantly different at *P* < 0.05 according to Tukey’s single-factor test. Data are shown as mean ± SD. At least three biological repetitions were performed.

## Supplementary Material

Web_Material_uhad188

## Data Availability

All data generated or analysed during this study are included in this published article and its supplementary information files. All of the required sequence files and annotation files of Chinese white pear were obtained from the Nanjing Agricultural University pear genome project website (http://peargenome.njau.edu.cn) [49]. The raw data that support the findings of this study have been deposited into CNGB Sequence Archive (CNSA: https://db.cngb.org/cnsa/) [50] of China National GeneBank DataBase (CNGBdb: https://db.cngb.org/) [51] with the project number CNP0003911.
