# Supplementary material for: PbrChiA: a key chitinase of pear in response to Botryosphaeria dothidea infection by interacting with PbrLYK1b2 and down-regulating ROS accumulation
Source: Hortic Res. 2023 Sep 19;10(10):uhad188. doi: 10.1093/hr/uhad188 (PMC10611555; doi:10.1093/hr/uhad188)
Supplement: Web_Material_uhad188 [file web_material_uhad188.zip › neo-FigS.pdf]

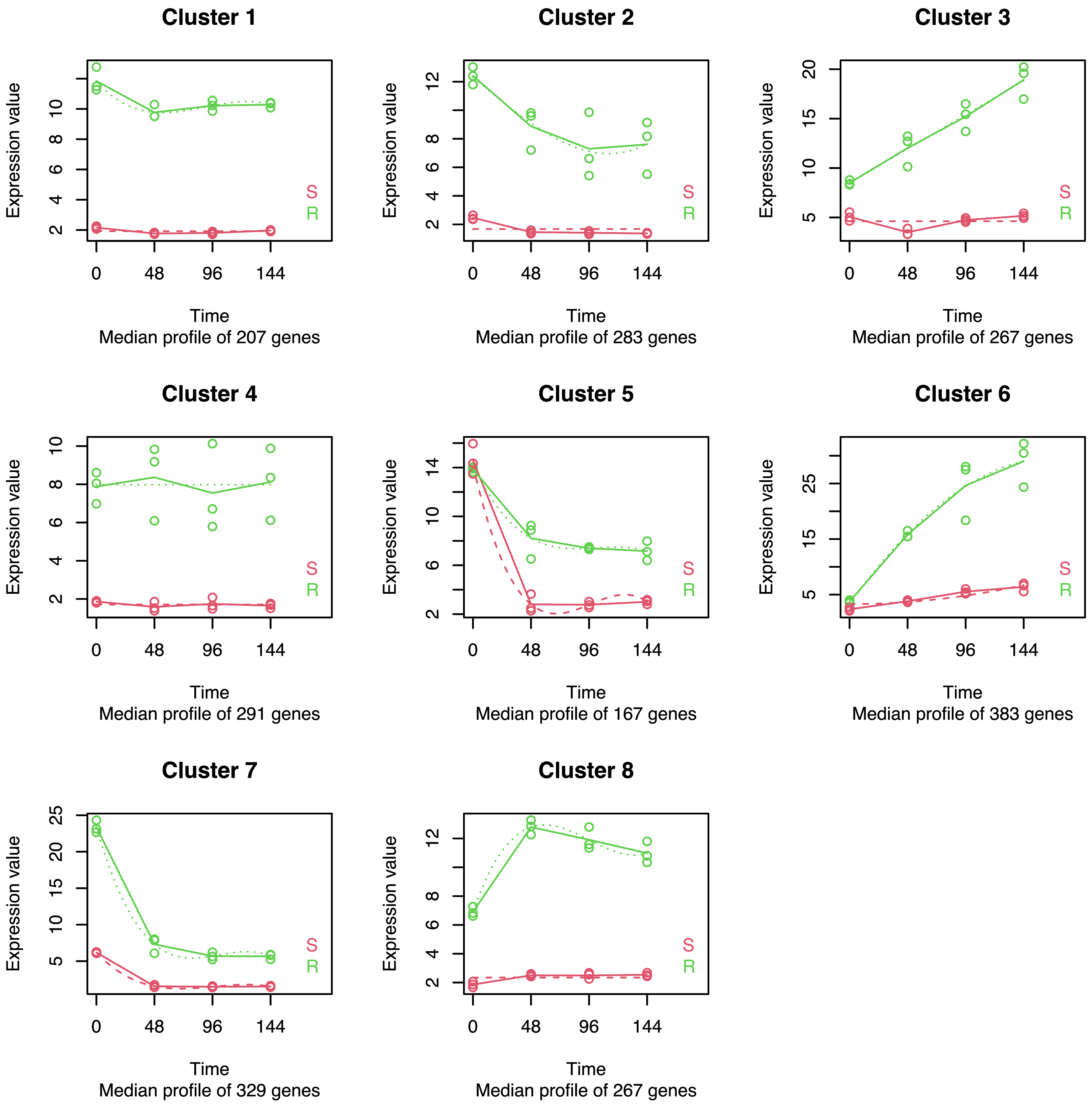
Cluster 1
Expression value
S
R
Time
Median profile of 207 genes
Cluster 2
Expression value
S
R
Time
Median profile of 283 genes
Cluster 3
Expression value
S
R
Time
Median profile of 267 genes
Cluster 4
Expression value
S
R
Time
Median profile of 291 genes
Cluster 5
Expression value
S
R
Time
Median profile of 167 genes
Cluster 6
Expression value
S
R
Time
Median profile of 383 genes
Cluster 7
Expression value
S
R
Time
Median profile of 329 genes
Cluster 8
Expression value
S
R
Time
Median profile of 267 genes
0
48
96
144

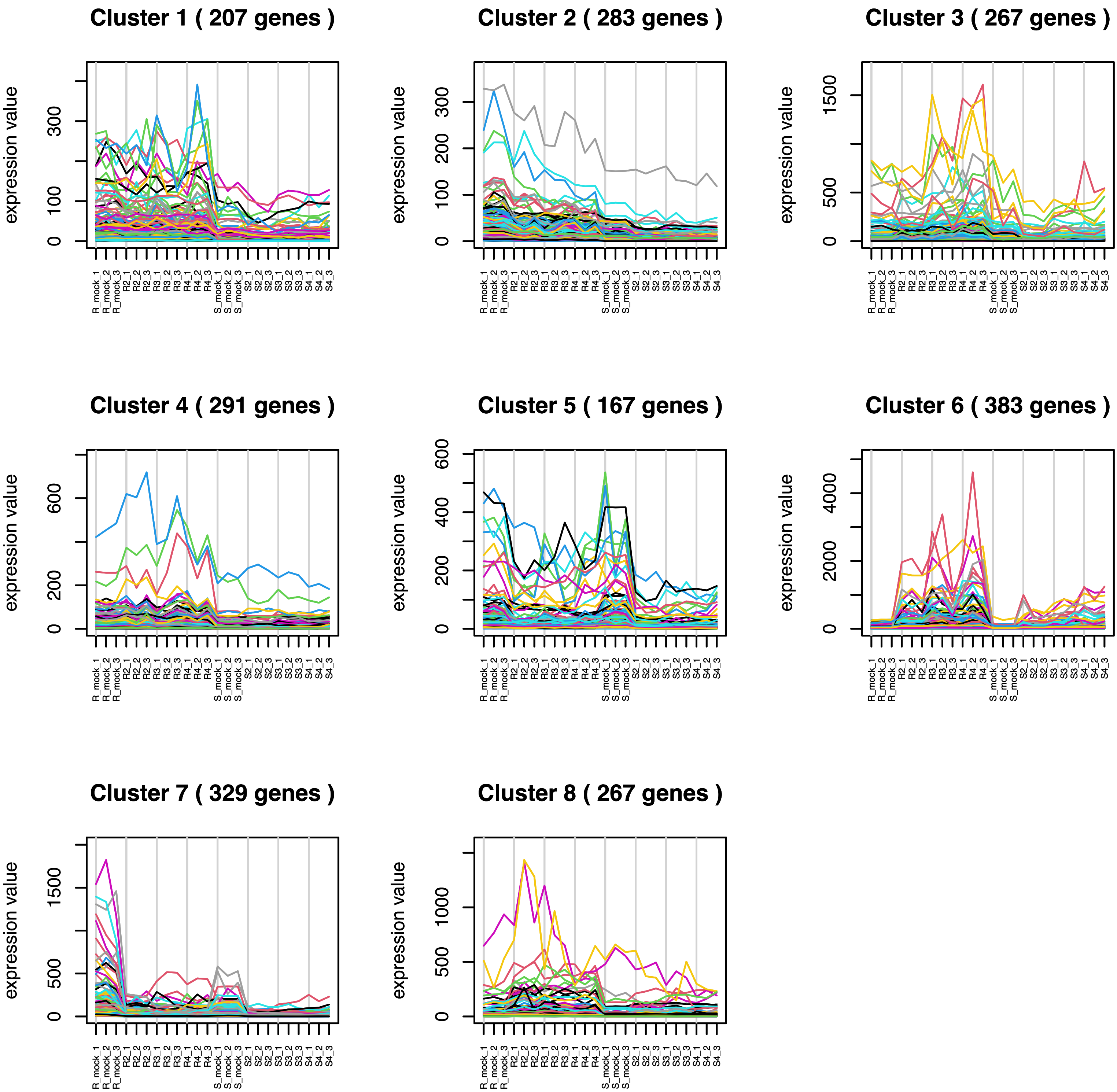

Cluster 1 (207 genes)
Cluster 2 (283 genes)
Cluster 3 (267 genes)
Cluster 4 (291 genes)
Cluster 5 (167 genes)
Cluster 6 (383 genes)
Cluster 7 (329 genes)
Cluster 8 (267 genes)
expression value
R_mock_1
R_mock_2
R_mock_3
R2_1
R2_2
R2_3
R3_1
R3_2
R3_3
R4_1
R4_2
R4_3
S_mock_1
S_mock_2
S_mock_3
S2_1
S2_2
S2_3
S3_1
S3_2
S3_3
S4_1
S4_2
S4_3

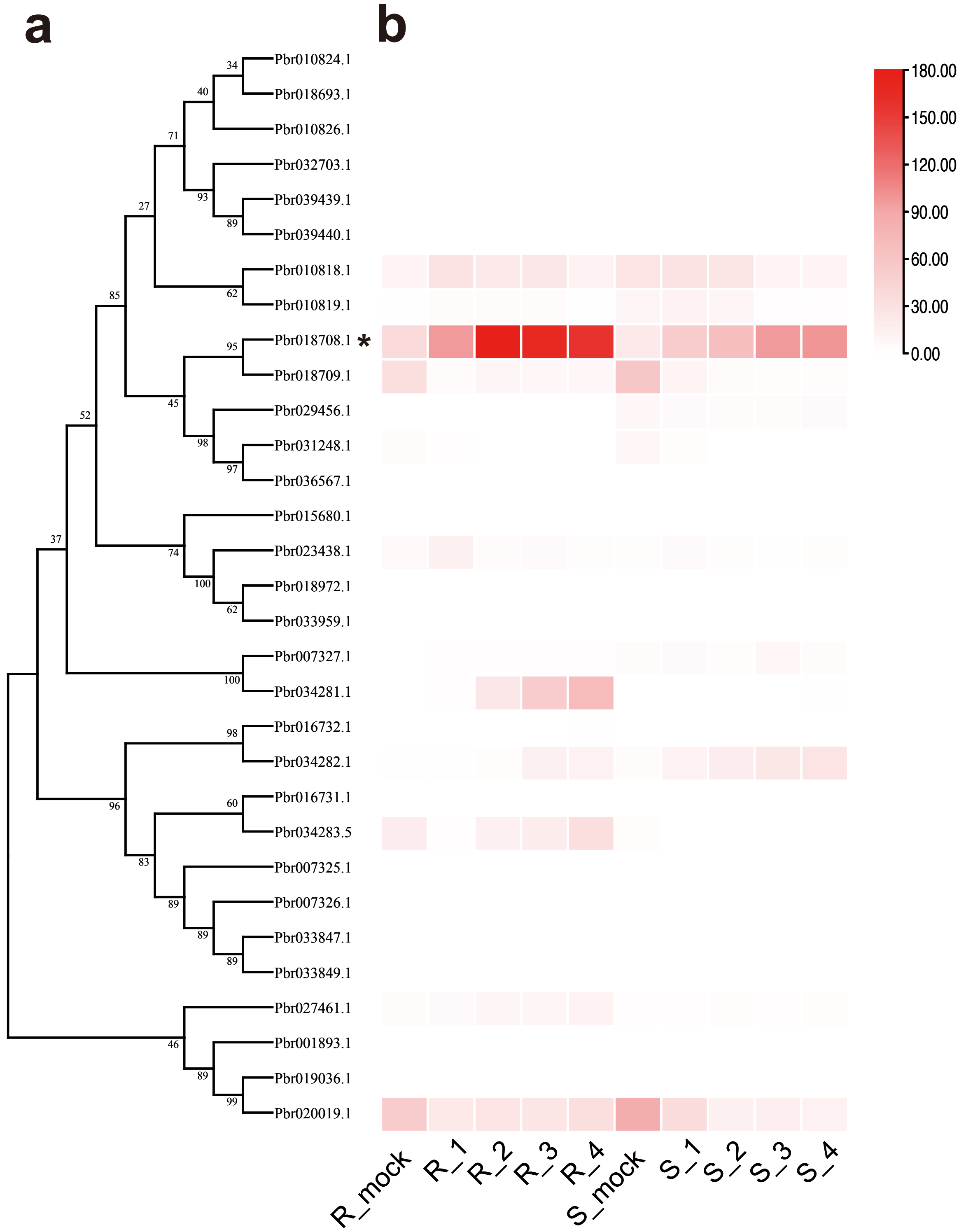

a
b
Pbr010824.1
Pbr018693.1
Pbr010826.1
Pbr032703.1
Pbr039439.1
Pbr039440.1
Pbr010818.1
Pbr010819.1
Pbr018708.1 *
Pbr018709.1
Pbr029456.1
Pbr031248.1
Pbr036567.1
Pbr015680.1
Pbr023438.1
Pbr018972.1
Pbr033959.1
Pbr007327.1
Pbr034281.1
Pbr016732.1
Pbr034282.1
Pbr016731.1
Pbr034283.5
Pbr007325.1
Pbr007326.1
Pbr033847.1
Pbr033849.1
Pbr027461.1
Pbr001893.1
Pbr019036.1
Pbr020019.1
34
40
71
93
89
27
62
85
95
45
98
97
52
37
74
100
62
100
98
96
60
83
89
89
89
46
89
99
R_mock
R_1
R_2
R_3
R_4
S_mock
S_1
S_2
S_3
S_4
180.00
150.00
120.00
90.00
60.00
30.00
0.00

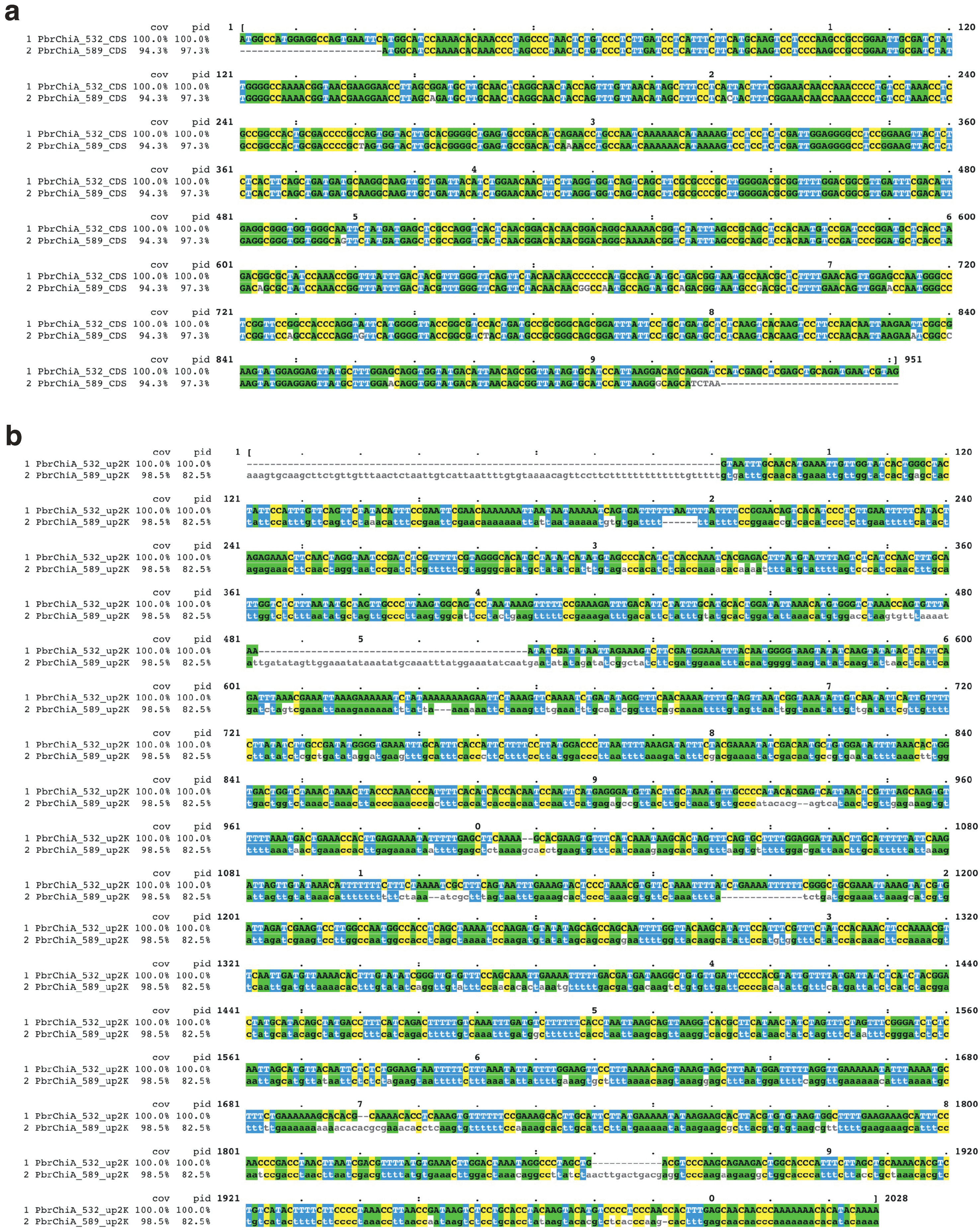
a
cov pid
1 PbrChiA_532_CDS 100.0% 100.0%
2 PbrChiA_589_CDS 94.3% 97.3%
b
cov pid
1 PbrChiA_532_up2K 100.0% 100.0%
2 PbrChiA_589_up2K 98.5% 82.5%

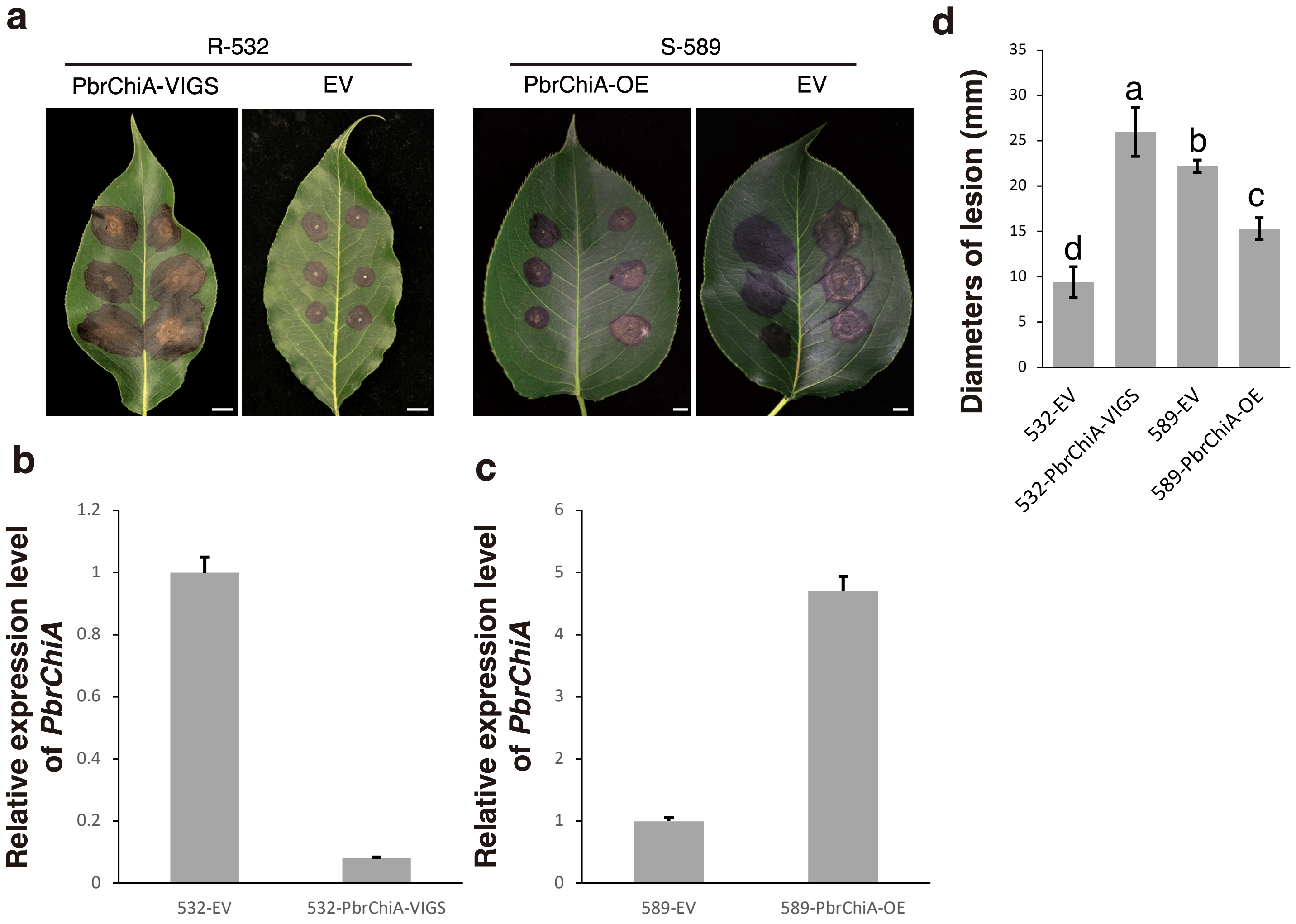
a
R-532
PbrChiA-VIGS
EV
S-589
PbrChiA-OE
EV
b
Relative expression level of *PbrChiA*
1.2
1
0.8
0.6
0.4
0.2
0
532-EV
532-PbrChiA-VIGS
c
Relative expression level of *PbrChiA*
6
5
4
3
2
1
0
589-EV
589-PbrChiA-OE
d
Diameters of lesion (mm)
35
30
25
20
15
10
5
0
a
b
c
d
532-EV
532-PbrChiA-VIGS
589-EV
589-PbrChiA-OE

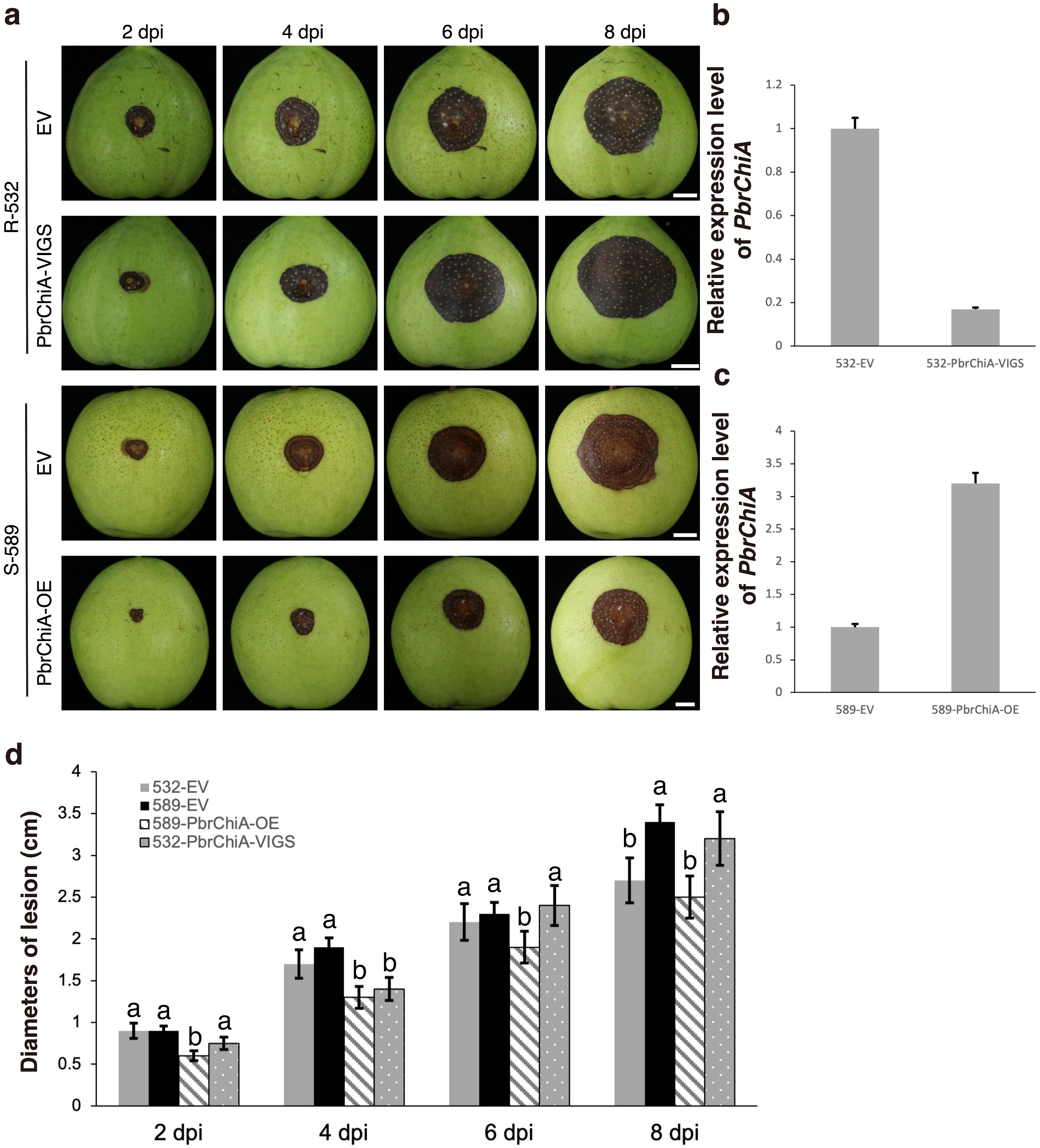
a
2 dpi
4 dpi
6 dpi
8 dpi
R-532
EV
PbrChiA-VIGS
S-589
EV
PbrChiA-OE
b
Relative expression level of PbrChiA
532-EV
532-PbrChiA-VIGS
c
Relative expression level of PbrChiA
589-EV
589-PbrChiA-OE
d
Diameters of lesion (cm)
532-EV
589-EV
589-PbrChiA-OE
532-PbrChiA-VIGS
2 dpi
4 dpi
6 dpi
8 dpi

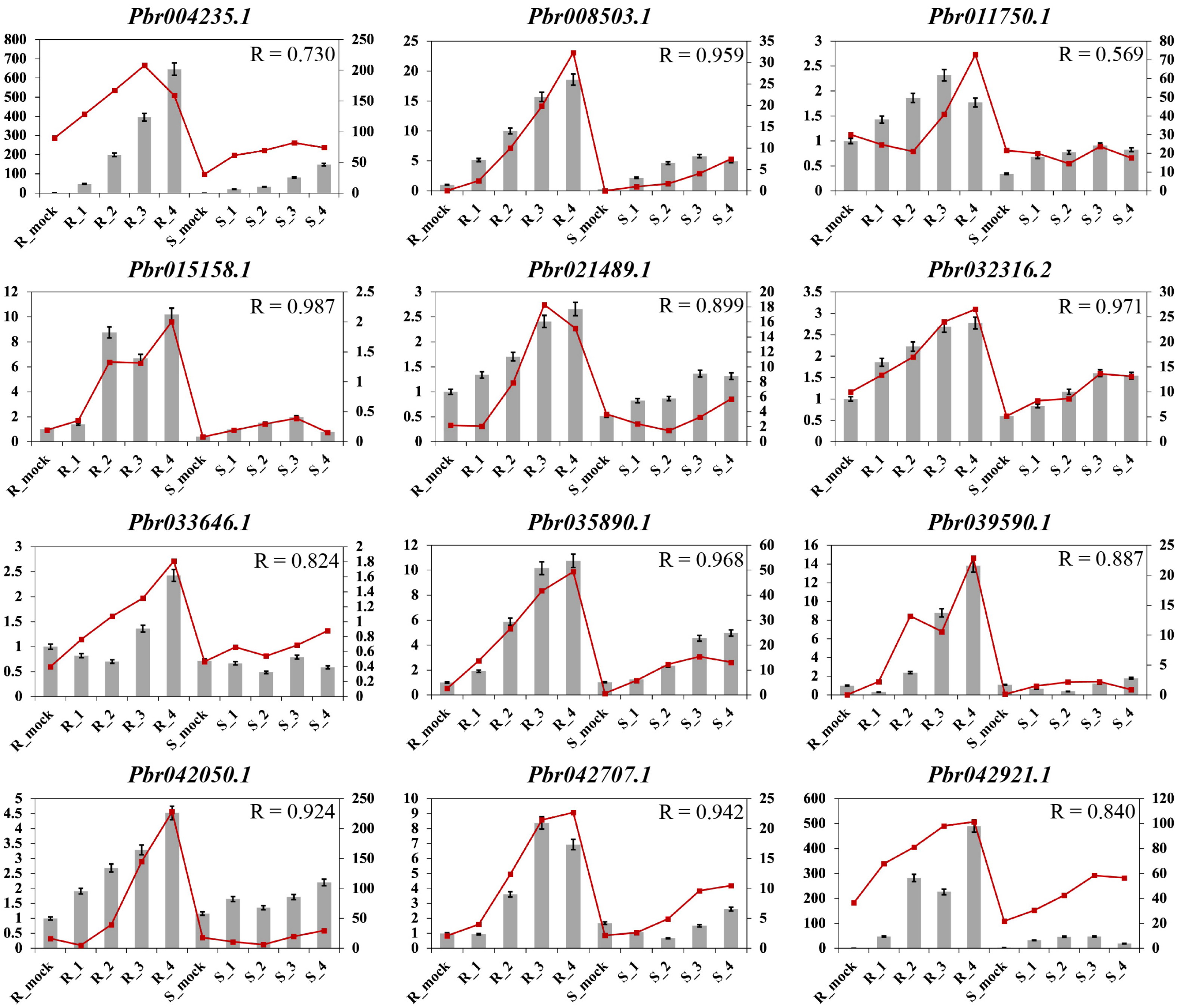

Pbr004235.1
R = 0.730
Pbr008503.1
R = 0.959
Pbr011750.1
R = 0.569
Pbr015158.1
R = 0.987
Pbr021489.1
R = 0.899
Pbr032316.2
R = 0.971
Pbr033646.1
R = 0.824
Pbr035890.1
R = 0.968
Pbr039590.1
R = 0.887
Pbr042050.1
R = 0.924
Pbr042707.1
R = 0.942
Pbr042921.1
R = 0.840
R_mock
R_1
R_2
R_3
R_4
S_mock
S_1
S_2
S_3
S_4

a

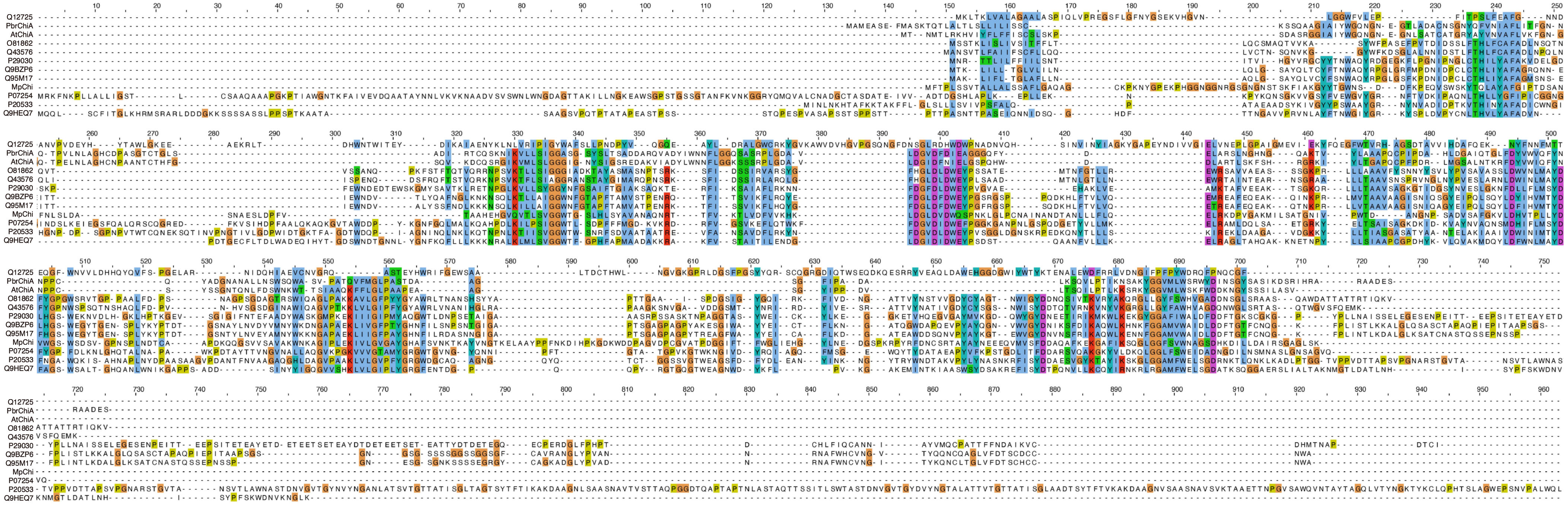
Q12725
PbrChiA
AtChiA
O81862
Q43576
P29030
Q9BZP6
Q95M17
MpChi
P07254
P20533
Q9HEQ7

b

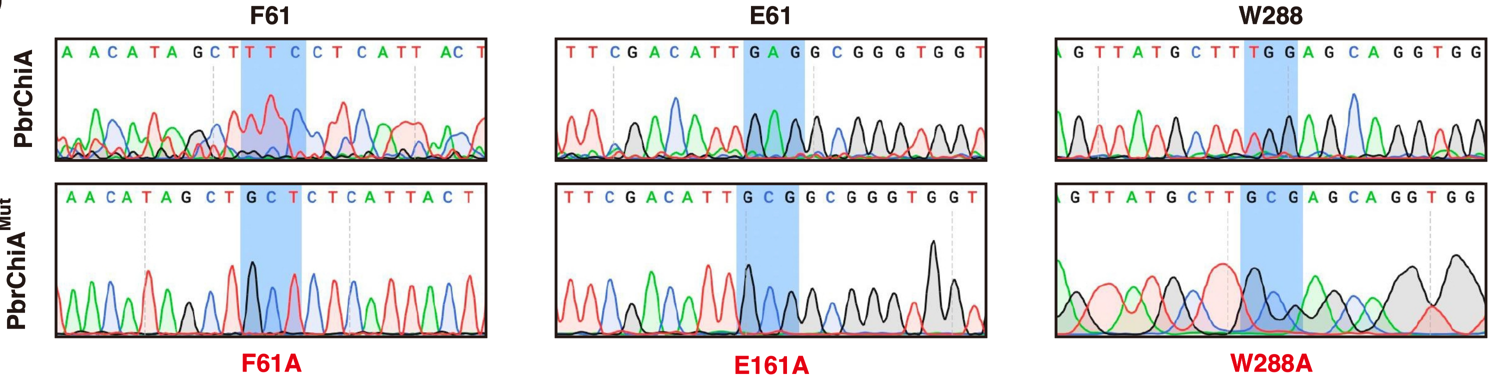
F61
E61
W288
PbrChiA
PbrChiA^Mut
A A C A T A G C T T T C C T C A T T A C T
T T C G A C A T T G A G G C G G G T G G T
G T T A T G C T T T G G A G C A G G T G G
A A C A T A G C T G C T C T C A T T A C T
T T C G A C A T T G C G G C G G G T G G T
G T T A T G C T T G C G A G C A G G T G G
F61A
E161A
W288A

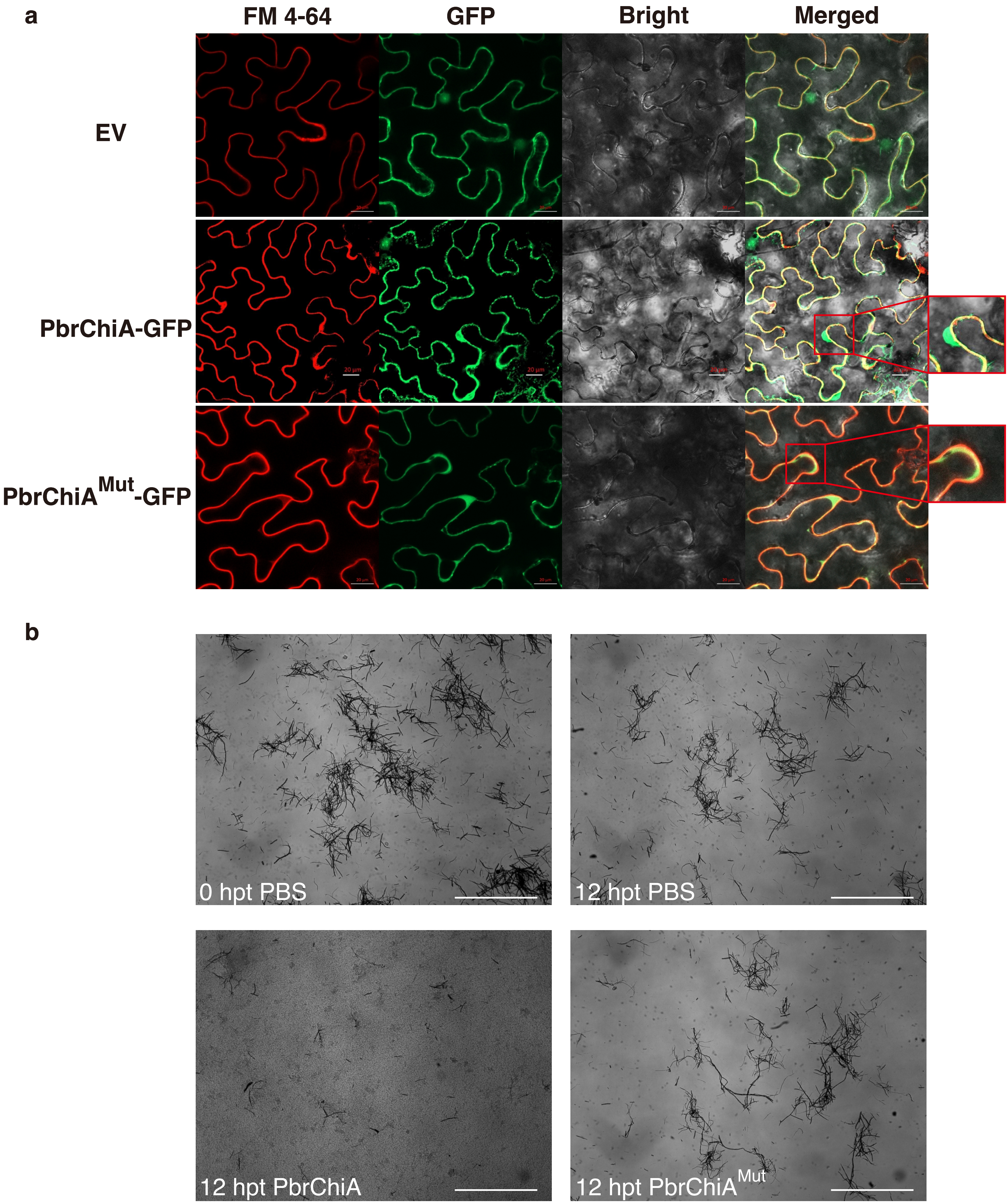
a
FM 4-64
GFP
Bright
Merged
EV
PbrChiA-GFP
PbrChiA^Mut-GFP
20 μm
b
0 hpt PBS
12 hpt PBS
12 hpt PbrChiA
12 hpt PbrChiA^Mut

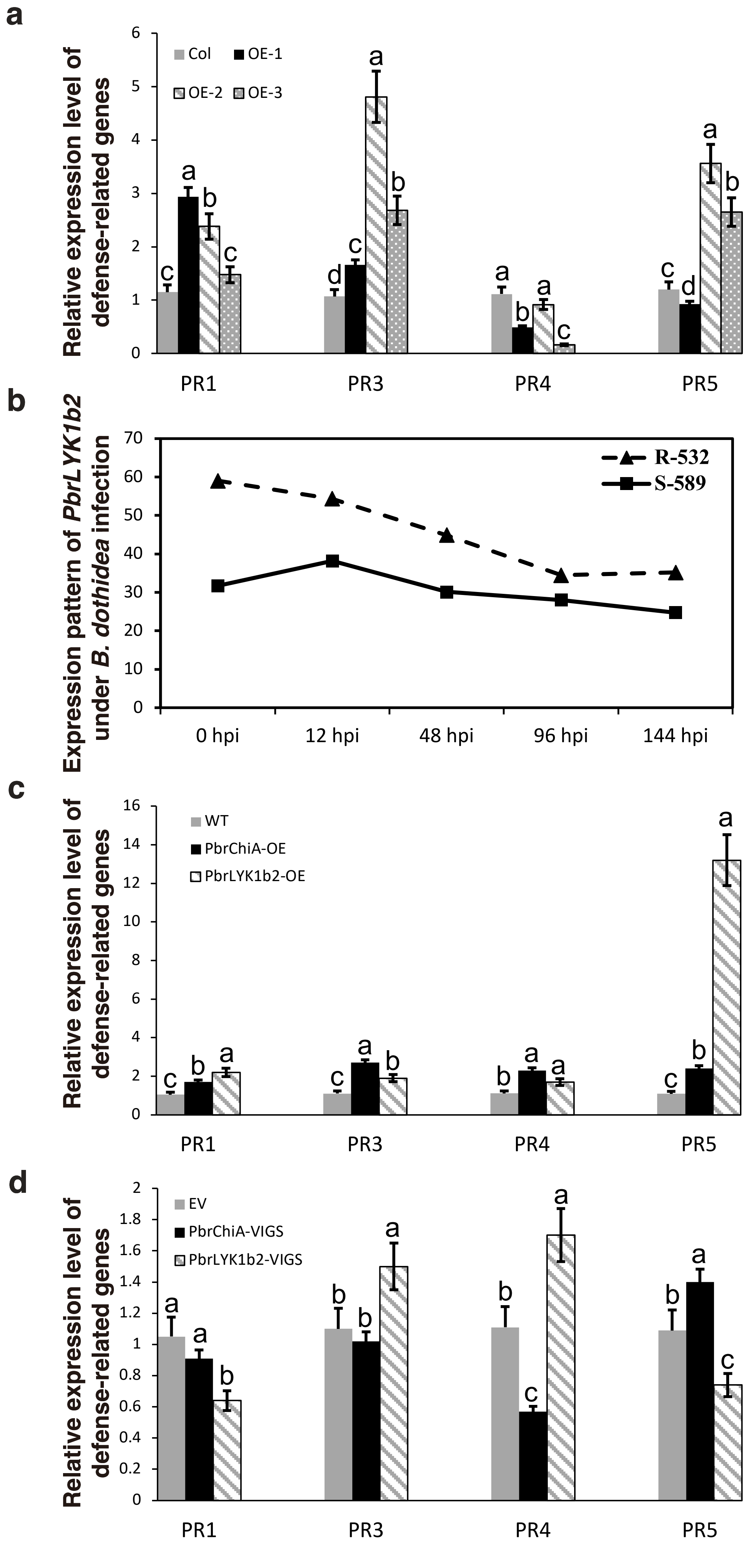
a
Relative expression level of defense-related genes
Col
OE-1
OE-2
OE-3
PR1
PR3
PR4
PR5
b
Expression pattern of PbrLYK1b2 under B. dothidea infection
R-532
S-589
0 hpi
12 hpi
48 hpi
96 hpi
144 hpi
c
Relative expression level of defense-related genes
WT
PbrChiA-OE
PbrLYK1b2-OE
PR1
PR3
PR4
PR5
d
Relative expression level of defense-related genes
EV
PbrChiA-VIGS
PbrLYK1b2-VIGS
PR1
PR3
PR4
PR5

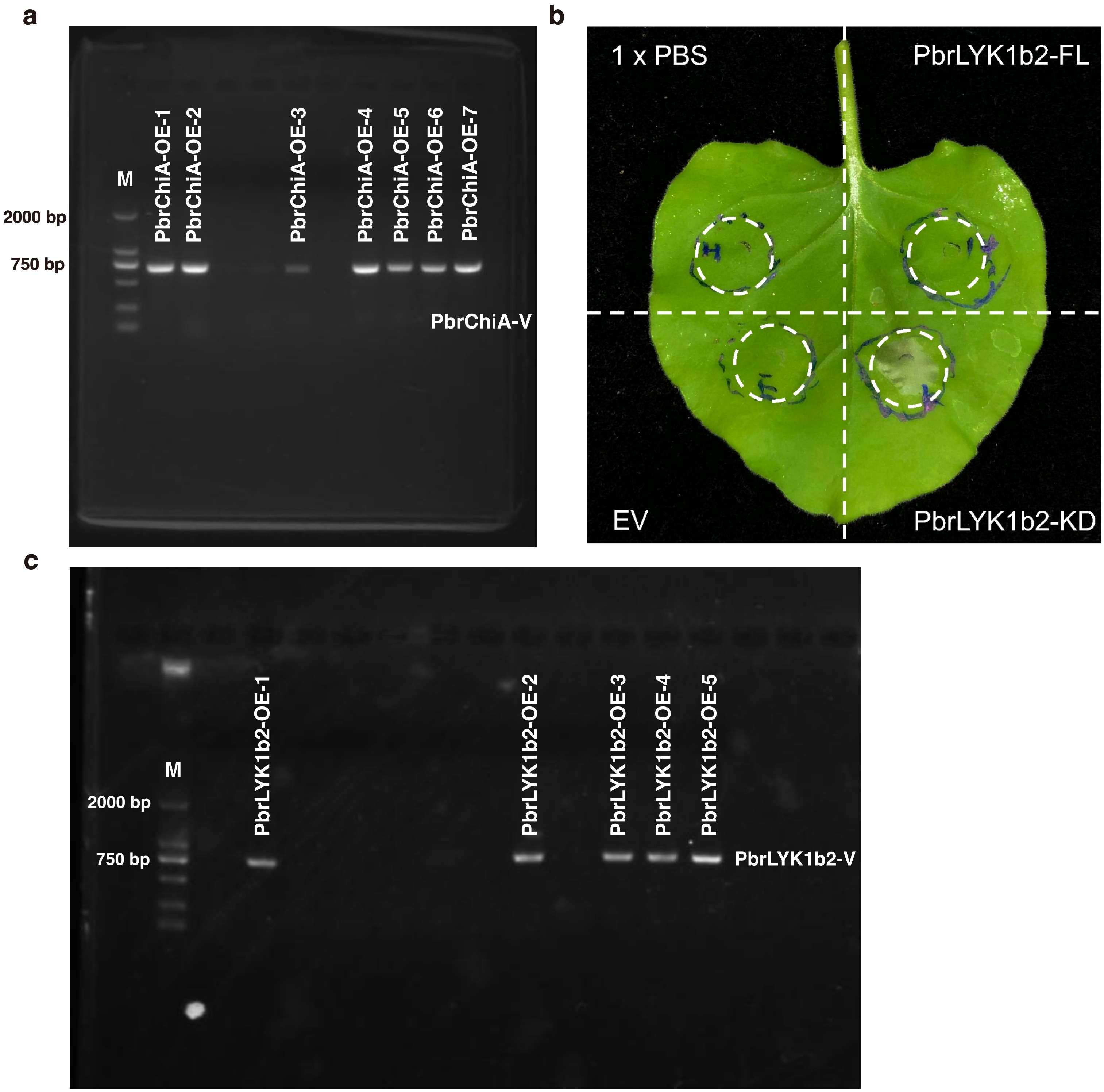

a
b
c
M
2000 bp
750 bp
PbrChiA-OE-1
PbrChiA-OE-2
PbrChiA-OE-3
PbrChiA-OE-4
PbrChiA-OE-5
PbrChiA-OE-6
PbrChiA-OE-7
PbrChiA-V
1 x PBS
PbrLYK1b2-FL
EV
PbrLYK1b2-KD
M
2000 bp
750 bp
PbrLYK1b2-OE-1
PbrLYK1b2-OE-2
PbrLYK1b2-OE-3
PbrLYK1b2-OE-4
PbrLYK1b2-OE-5
PbrLYK1b2-V

WT

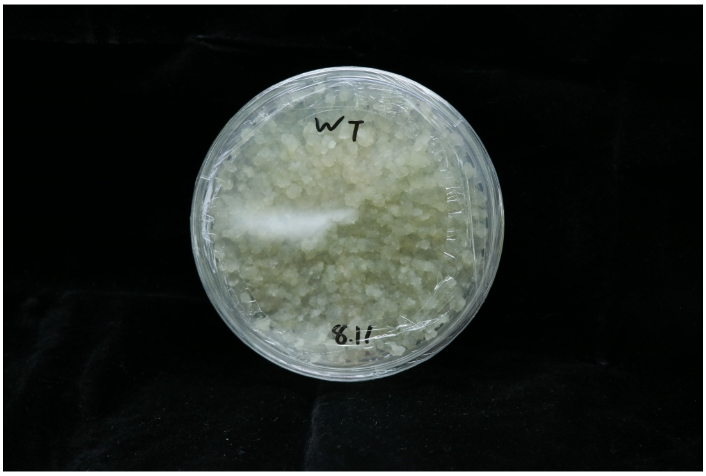

PbrChiA-OE

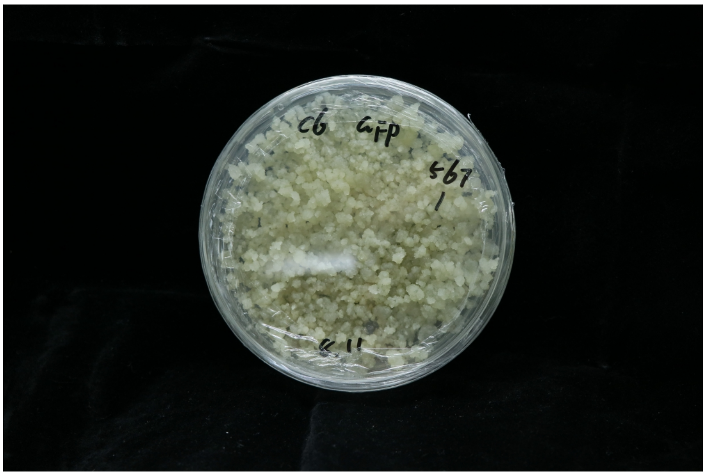

PbrLYK1b2-OE

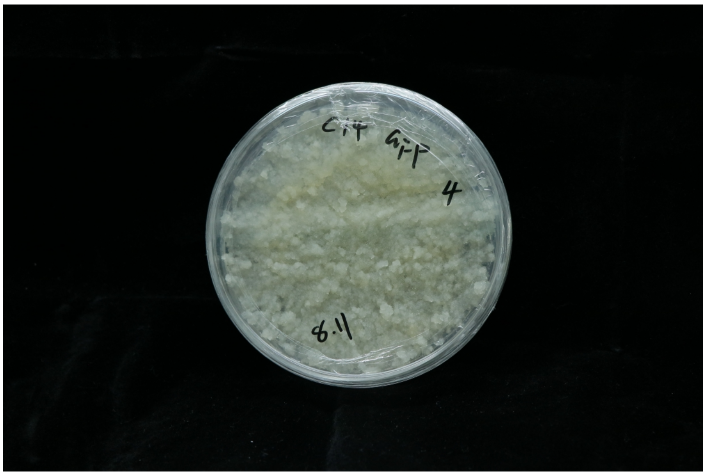

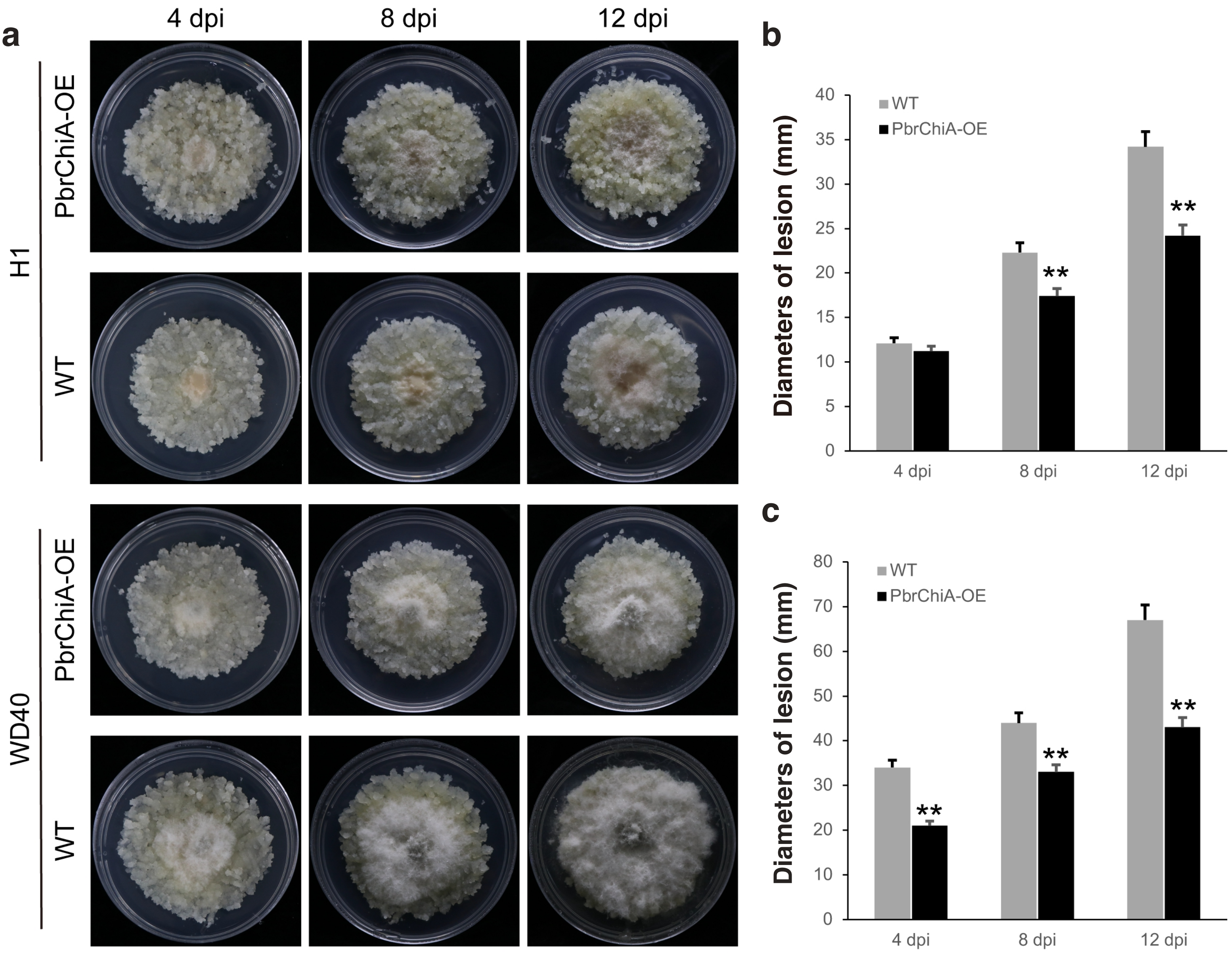

a
4 dpi
8 dpi
12 dpi
H1
PbrChiA-OE
WT
WD40
PbrChiA-OE
WT
b
WT
PbrChiA-OE
Diameters of lesion (mm)
40
35
30
25
20
15
10
5
0
**
**
4 dpi
8 dpi
12 dpi
c
WT
PbrChiA-OE
Diameters of lesion (mm)
80
70
60
50
40
30
20
10
0
**
**
**
4 dpi
8 dpi
12 dpi

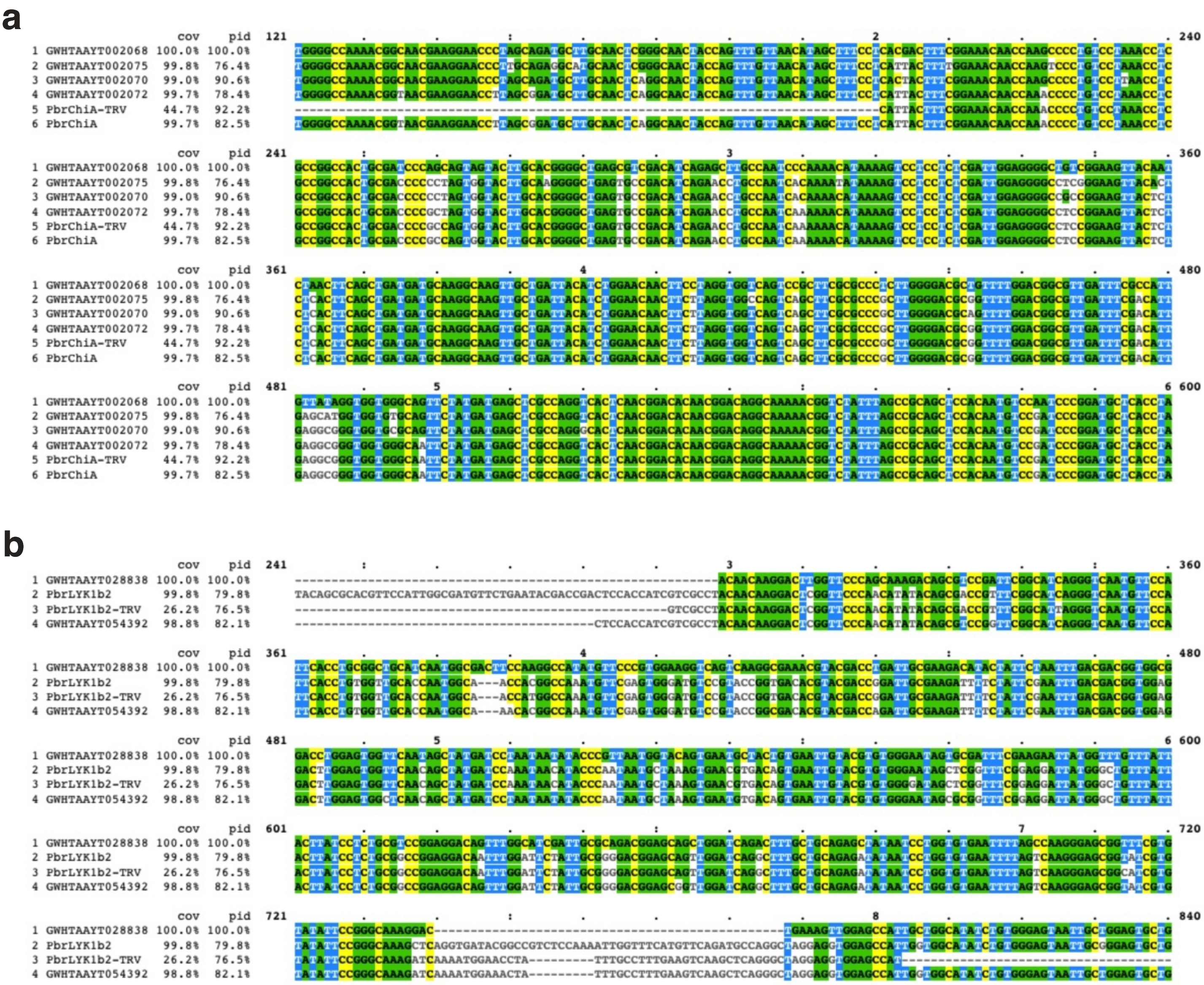
a
cov pid
1 GWHTAAYT002068 100.0% 100.0%
2 GWHTAAYT002075 99.8% 76.4%
3 GWHTAAYT002070 99.0% 90.6%
4 GWHTAAYT002072 99.7% 78.4%
5 PbrChiA-TRV 44.7% 92.2%
6 PbrChiA 99.7% 82.5%
b
cov pid
1 GWHTAAYT028838 100.0% 100.0%
2 PbrLYK1b2 99.8% 79.8%
3 PbrLYK1b2-TRV 26.2% 76.5%
4 GWHTAAYT054392 98.8% 82.1%

a

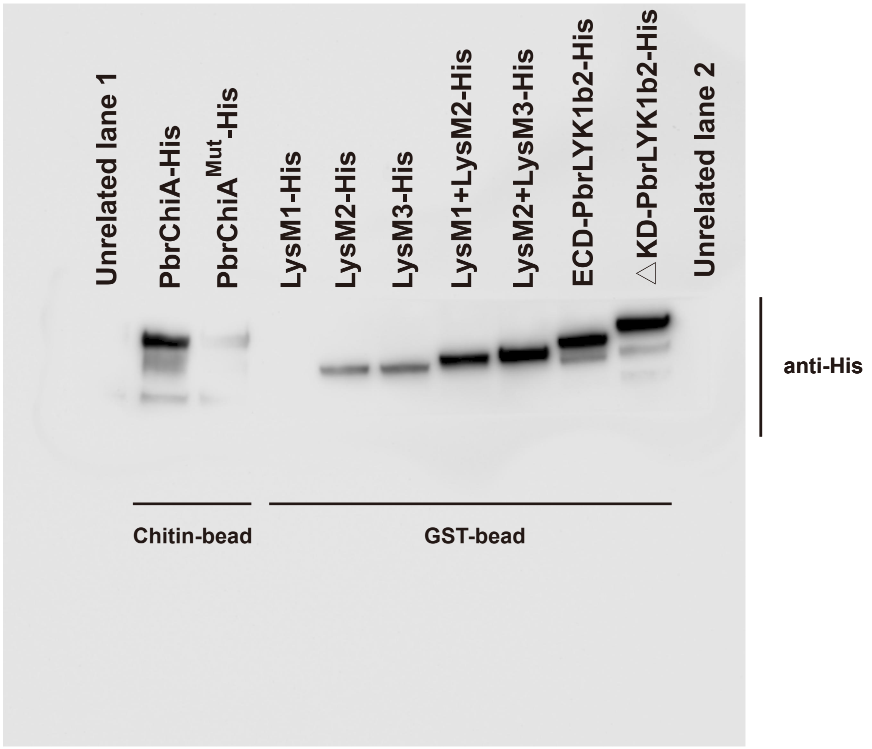
Unrelated lane 1
PbrChiA-His
PbrChiA^Mut-His
LysM1-His
LysM2-His
LysM3-His
LysM1+LysM2-His
LysM2+LysM3-His
ECD-PbrLYK1b2-His
△KD-PbrLYK1b2-His
Unrelated lane 2
anti-His
Chitin-bead
GST-bead

b

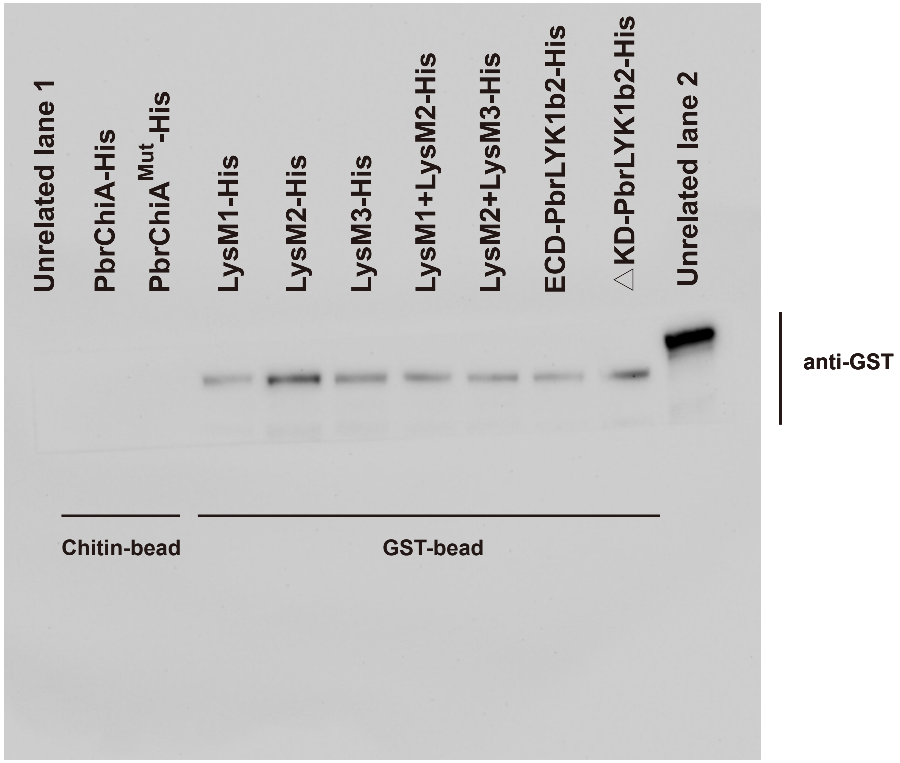
Unrelated lane 1
PbrChiA-His
PbrChiA^Mut-His
LysM1-His
LysM2-His
LysM3-His
LysM1+LysM2-His
LysM2+LysM3-His
ECD-PbrLYK1b2-His
△KD-PbrLYK1b2-His
Unrelated lane 2
anti-GST
Chitin-bead
GST-bead
